# Linking Genotype and Phenotype of *Saccharomyces cerevisiae* Strains Reveals Metabolic Engineering Targets and Leads to Triterpene Hyper-Producers

**DOI:** 10.1371/journal.pone.0014763

**Published:** 2011-03-18

**Authors:** Karina M. Madsen, Gupta D. B. R. K. Udatha, Saori Semba, Jose M. Otero, Peter Koetter, Jens Nielsen, Yutaka Ebizuka, Tetsuo Kushiro, Gianni Panagiotou

**Affiliations:** 1 Graduate School of Pharmaceutical Sciences, The University of Tokyo, Hongo, Bunkyo-ku, Tokyo, Japan; 2 Center for Microbial Biotechnology, Department of Systems Biology, Technical University of Denmark, Kgs. Lyngby, Denmark; 3 Institute for Microbiology, Johann Wolfgang Goethe-University of Frankfurt, Frankfurt, Germany; 4 Department of Chemical and Biological Engineering, Systems Biology, Chalmers University of Technology, Gothenburg, Sweden; 5 Department of Chemical and Biological Engineering, Industrial Biotechnology, Chalmers University of Technology, Gothenburg, Sweden; 6 Center for Biological Sequence Analysis, Department of Systems Biology, Technical University of Denmark, Lyngby, Denmark; King's College London, United Kingdom

## Abstract

**Background:**

Metabolic engineering is an attractive approach in order to improve the microbial production of drugs. Triterpenes is a chemically diverse class of compounds and many among them are of interest from a human health perspective. A systematic experimental or computational survey of all feasible gene modifications to determine the genotype yielding the optimal triterpene production phenotype is a laborious and time-consuming process.

**Methodology/Principal Findings:**

Based on the recent genome-wide sequencing of *Saccharomyces cerevisiae* CEN.PK 113-7D and its phenotypic differences with the S288C strain, we implemented a strategy for the construction of a β-amyrin production platform. The genes *Erg8*, *Erg9* and *HFA1* contained non-silent SNPs that were computationally analyzed to evaluate the changes that cause in the respective protein structures. Subsequently, *Erg8, Erg9* and *HFA1* were correlated with the increased levels of ergosterol and fatty acids in CEN.PK 113-7D and single, double, and triple gene over-expression strains were constructed.

**Conclusions:**

The six out of seven gene over-expression constructs had a considerable impact on both ergosterol and β-amyrin production. In the case of β-amyrin formation the triple over-expression construct exhibited a nearly 500% increase over the control strain making our metabolic engineering strategy the most successful design of triterpene microbial producers.

## Introduction

Metabolic engineering, which integrates engineering design with systematic and quantitative analysis of metabolic pathways, is considered as one of the major concepts in biotechnology [1]. The central goal of metabolic engineering is the optimization of the metabolic phenotype with an emphasis on the global state of the cell, and not the individual reactions [2]. This manipulation of the system with consideration of the efficiency of the overall bioprocess is what distinguishes metabolic engineering from genetic engineering [3]. Well-characterized and genetically fairly easy to manipulate heterologous hosts, like *Escherichia coli* and *Saccharomyces cerevisiae*, allow very specific engineering of biosynthetic pathways for increased yields and generation of novel compounds. After engineering a pathway, it is desirable to analyze the metabolic profile to be able to compare before and after situations and detect effects on the pathway originating from distant networks [4].

Metabolic engineering of microorganisms through the expression of one or more plant genes, often in connection with genetic alteration of the whole cell metabolism, has become an increasingly important route for small molecule synthesis. Terpenoids, with more than 55,000 members identified, have particularly benefited from this approach [5]. The value of these natural products extends beyond their biological utility and they have been commercialized to serve as antibiotics, anticancer and other medicinal products. The need for metabolic engineering as a framework of terpenoid production has arisen mainly as a result of supply issues, since these molecules are synthesized in only minute amounts in the natural hosts hampering their commercialization. Engineering of plant terpenoids into microbial hosts has been focused primarily on isoprenoid-derived compounds such as carotenoids, artemisin, and paclitaxel [6]-[8].

**Figure 1:**
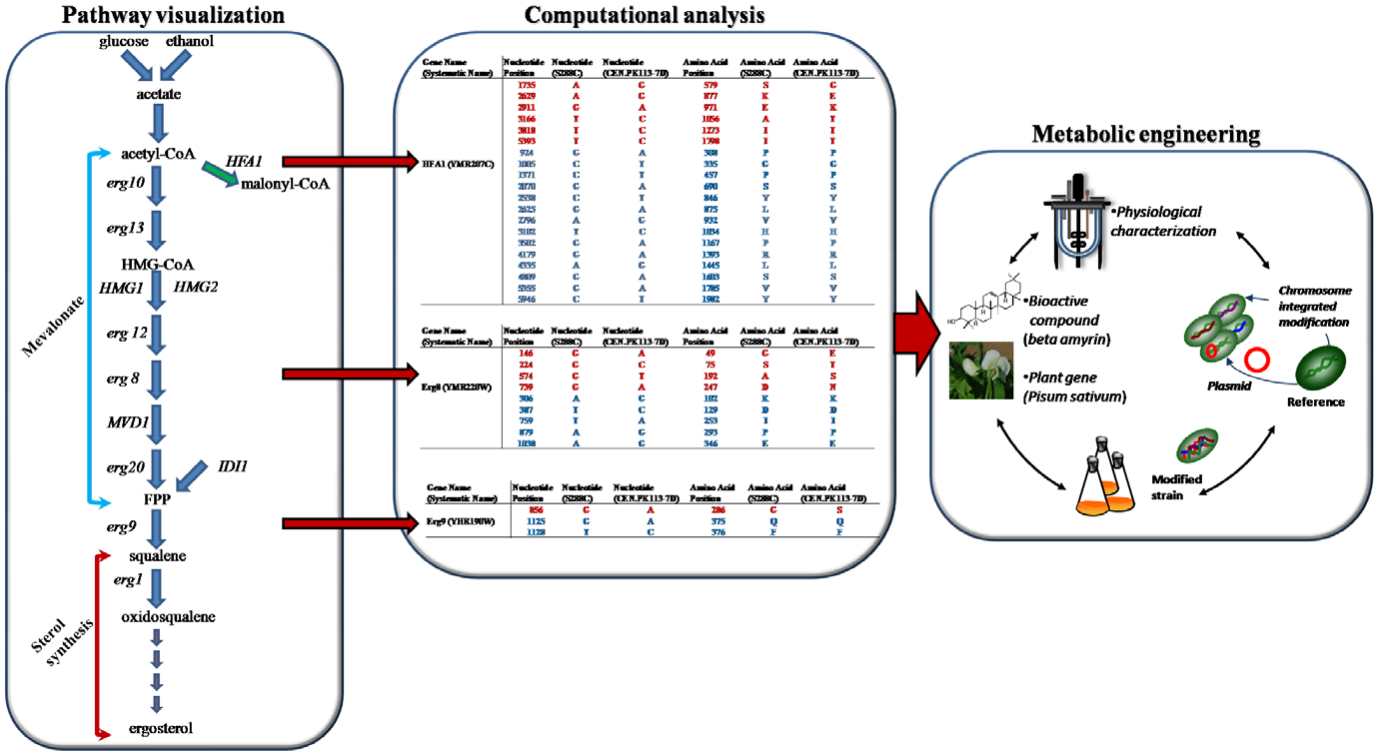
Schematic illustration of the mevalonate, the sterol pathway and the initial step of the fatty acid biosynthetic process, as well as the steps engineered in the current study for triterpene production in yeast. The mevalonate pathway is localized to the cytoplasm of eukaryotic cells and supports the biosynthesis of numerous terpenoids using different precursor molecules, while ergosterol is the dominant terpenoid. Whole genome Illumina-Solexa sequencing of CEN.PK113-7D and S288C was completed prior to our study, and SNPs strictly related to metabolic genes were identified [12]. There were clear correlations between physiology and pathway enrichment of non-silent SNPs observed in genes involved in the ergosterol biosynthesis (red font indicates non-silent SNPs, while blue font indicates silent ones), suggesting that genome-sequencing may assist in reducing the genetic target space for metabolic engineering applications. Various combinations of over-expressions (single, double, triple) of genes coding for phosphomevalonate kinase (*Erg8*), squalene synthase (*Erg9*), and acetyl-coenzyme A carboxylase (*HFA1*) may yield yeast strains capable of accumulating excess levels of β-amyrin, a triterpene molecule originating from oxidosqualene.

Metabolic pathways are stamped by natural bottlenecks, which serve as control points within a native organism, to regulate resource utilization and production of metabolites. The ultimate goal of metabolic engineering is to predict the engineering required for increasing (or maximizing) a metabolic flux through a desired pathway, however, this has been particularly challenging [9]. The lack of extensive knowledge about molecular interactions and their kinetics makes the dissection and optimization of metabolic pathways an outstanding issue of central importance [10]. The identification of distant genes affecting a metabolic phenotype, either through redistribution of metabolic precursors or indirect kinetic and global regulatory effects recently spurred by the high-throughput ‘omics’ and genome-based bioinformatic approaches. Here we describe a novel method for pathway optimization that focuses on identifying rate-limiting enzymes. We establish a proof-of-concept that whole genome sequencing can be used to identify single nucleotide polymorphisms between *S. cerevisiae* strains, which can be subsequently linked with particular phenotypes of interest (Figure 1). For example Daum *et al* [11] have observed that the content of ergosterol and fatty acids in CEN.PK is significantly higher than other yeast strains indicating a possible correlation between genotype and phenotype. Previously to our work, a total of 13,787 high-quality SNPs, of which 782 in metabolic genes [12], were detected when the CEN.PK 113-7D sequence was compared to the S288C, the reference genome of the *Saccharomyces* Genome Database. In the genomic comparisons of the two strains by Otero *et al* the ergosterol biosynthetic pathway had several non-silent SNPs identified in *Erg8* and *Erg9*, and silent SNPs identified in *Erg20* and *HMG1* [12]. In their paper the authors performed also a transcriptome comparison between the two yeast strains. Both *Erg8* and *Erg9* were not significantly differentially expressed in glucose suggesting that their potential affect on phenotype is likely post-transational. Amino acid substitutions resulting from SNPs can enhance the properties of a protein such as stability or catalytic activity and are essential raw material of evolution [13]. They are starting points for the adaptive evolution of new functions and often occur through pathways consisting of sequential beneficial mutations [14]. The effect of mutations on stability (ΔΔ*G*) of proteins has been explored by several researchers [15]-[17]. It has been shown that mutated proteins that are more stable than a particular threshold energy can fold properly and result in improved or changed function [18]-[21]. Changes in inter-residue interactions caused by mutations are also important for understanding protein folding and stability patterns of proteins [22].

The current work was undertaken to develop *S. cerevisiae* as a production platform of triterpenoids using direct correlations between genotype and phenotype. We describe here the utilization of detected metabolic SNPs for constructing 7 yeast mutants engineered to enhance carbon flux through the mevalonate pathway and accumulate high levels of β-amyrin (Figure 1). Such developments support long range objectives to generate large quantities of end-product triterpenoids sufficient for detailed chemical analyses and diverse biological and industrial testing.

## Results

### SNPs role on *Erg8*, *Erg9* and *HFA1*

In their paper on the genome wide sequencing of CEN.PK113-7D, Otero *et al* [12] identified two pathways with a significant number of SNPs (Figure 1), both silent (blue font) and non-silent (red font). *Erg8* and *Erg9*, both participating in the ergosterol biosynthesis pathway, included in total 7 silent and 5 non-silent SNPs. In *Erg8* there were detected 4 non silent SNPs in positions 49, 75, 192, and 247, while in *Erg9* there was only 1 non silent SNP in position 286 (Figure 1). In fatty acid metabolism, the *HFA1* gene was highly enriched with 20 silent and non-silent SNPs. *HFA1* contained non-silent SNPs in amino acid positions 579, 877, 971, 1056, 1273, and 1798.

**Figure 2:**
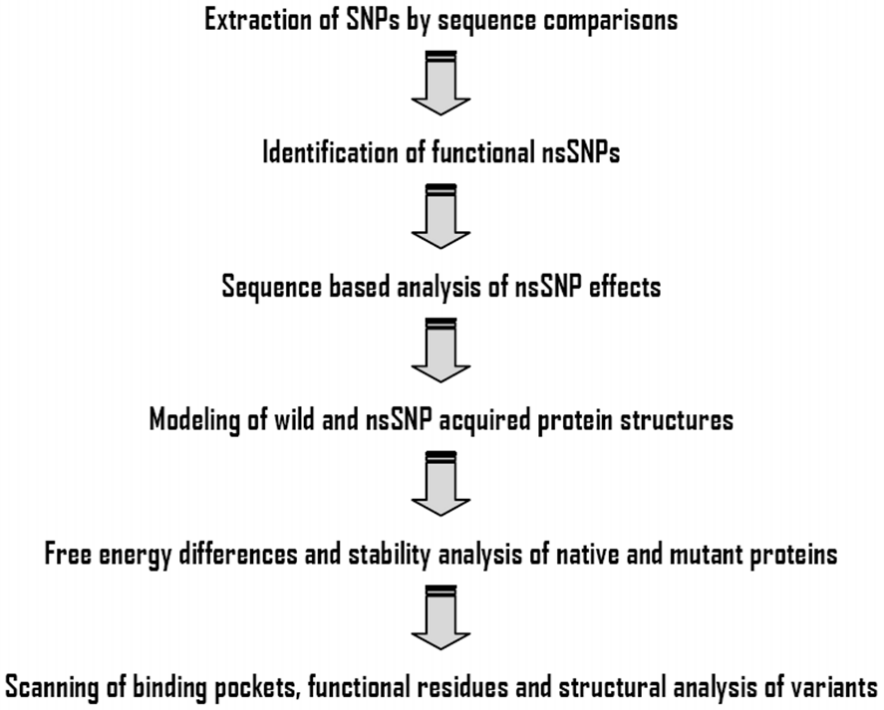
Strategy for computational analysis of the non-silent (ns)SNPs.

Predicting the effects of the nsSNPs on the protein structure-stability-function of Erg8, Erg9 and HFA1 is very important for selecting the three genes as metabolic engineering targets. The computational strategy shown in Figure 2 was employed in the present study, however our findings should be evaluated with caution since no experimental verification was obtained for the three last steps of the flowchart.

### Computational analysis of nsSNPs based on primary amino acid sequence

The underlying principle of the SIFT algorithm is that it generates alignments with a large number of homologous sequences and assign a tolerance index score to each amino acid substitution ranging from 0 to 1 [23]. The higher the tolerance index of a mutant is, the less functional impact the respective amino acid substitution is likely to have. The results of SIFT for respective amino substitutions in *Erg8*, *Erg9* and *HFA1* sequences are shown in Supporting Information S1. The results were examined by considering the *S. cerevisiae* S288C as the “wild type strain” and the CEN.PK113-7D as the “mutant strain” in the first step, and vice versa. From the SIFT scores, it appears that the nsSNPs of Erg8 and HFA1 have an overall effect on CEN.PK113-7D, whereas, the nsSNP of Erg9 has a neutral effect. This assessment of nsSNPs by SIFT is mainly based on the conserved positions along the amino acid sequences, and to understand the effect of nsSNPs on inter-residue interactions and protein stability, structural analysis is a necessity.

### Protein stability estimation in coding nsSNPs based on 3D structures

Sequence and secondary structure alignments of phosphomevalonate kinase (the protein product of *Erg8*) against the protein models by PMP resulted into the selection of the Lin0012 protein from *Listeria innocua* (DOI:10.2210/pdb3k17/pdb) as a template for homology modeling. The secondary structure alignments and respective scores generated using ClustalW [24] are shown in Supporting Information S1. Pairwise structural alignment of the 3k17C and the *Erg8* protein product was employed using the Dali server [25] and is shown in File S1. The 3D-structural similarity information of the Lin0012 protein from *Listeria innocua* (PDB ID: 3k17) with the existing crystal structures was retrieved from the Research Collaboratory for Structural Bioinformatics – Protein Data Bank [26] that uses the FATCAT method for flexible structural alignments of proteins. The information shown in Supporting Information S1 depicts the similarity of Lin0012 protein from *Listeria innocua* with kinase enzymes. The homology model validation (Supporting Information S1) of the *Erg8* protein products from *S. cerevisiae* S288C and CEN.PK113-7D using the ProSA-web showed *z*-scores of -6.1 and -5.97, respectively. The *z*-scores of homology models for both *Erg8* protein products are in the range characteristic for x-ray determined structures deposited in Protein Data Bank.

The homology modeling and structure validation of the *Erg9* protein products from *S. cerevisiae* S288C and CEN.PK113-7D was performed as above. Sequence and secondary structure alignments for squalene synthase against the protein models by PMP resulted into the selection of a human squalene synthase [27] as a template. The secondary structure alignments and respective scores generated using ClustalW [24] are also shown in Supporting Information S1. Pairwise structural alignment of the 1ezfC and the *Erg9* protein product was employed using the Dali server [25] and it is shown in Supporting Information S1. The homology model validation (Supporting Information S1) of the *Erg9* protein products from *S. cerevisiae* S288C and CEN.PK113-7D using the ProSA-web showed *z*-scores of -7.88 and -7.85, respectively.

Yeast contains two distinct acetyl-CoA carboxylase multi-component enzyme systems, one in the cytoplasm encoded by *ACC1*, and another one in the mitochondrial matrix encoded by *HFA1* [28]. The *HFA1* protein product consists of three functional units (Supporting Information S1), biotin carboxylase, biotinoyl domain or biotin-carboxyl-carrier protein and carboxyl transferase [29]. The homology models of the *HFA1* protein product catalytic domains were built using MODWEB, which is based on MODPIPE, an automated software pipeline for comparative modeling [30]-[31]. The modelled segments of the HFA1 protein and the respective templates used for homology modeling are shown in Supporting Information S1. Out of six nsSNPs, only one nsSNP leading to amino acid substitution (I1798T) fall in a catalytic domain of the *HFA1* protein product, i.e., carboxyl transferase domain. Therefore, the carboxyl transferase domain was considered for further analysis. The homology model validation (Supporting Information S1) for the carboxyl tranferse domain of the *HFA1* protein products from *S. cerevisiae* S288C and CEN.PK113-7D using ProSA-web showed *z*-scores of -8.51 and -8.53, respectively.

The change in the protein stability (ΔΔG) induced by mutations calculated by the Eris server indicated that *Erg8, Erg 9* and *HFA1* protein products from *S. cerevisiae* CEN.PK113-7D were probably more stable than that from S288C (Supporting Information S2). Accessible Surface Area calculations for the Erg8, Erg9 and HFA1 proteins and respective energies calculated by the InterProPatch server (Supporting Information S2) also strengthen the protein stability predictions obtained from Eris.

### Graph theoretic measures of structural effects in proteins caused by individual nsSNPs

*Bongo* calculates the overall impact (*I*) of a mutation according to the ‘key’ residues affected by the mutation [32]. To understand the notation of ‘key’ residues, let's consider the amino acid substitutions of the *Erg8* protein product. Comparison of residue-residue interaction graphs (Supporting Information S2) clearly shows that amino acid substitutions viz., G49E, S75T and D247N have no change in local environment of interactions with other residues, whereas A129S amino acid substitution changes both local and global residue-residue interaction networks. Analysis of the effect of individual nsSNPs by *Bongo* shows that G49E, S75T and D247N amino acid substitutions have an overall impact value within the threshold (*I*<1), whereas A129S amino acid substitution shows an impact value greater than 1 (*I*>1) and therefore may cause structural effects on the *Erg8* protein product. A protein can tolerate functionally beneficial but destabilizing substitutions, only if it has previously acquired one or more stabilizing mutations [33]. In the case of the *Erg9* protein product, the nsSNP or amino acid substitution G286S appears to have no effect on local or global residue-residue interaction networks (Supporting Information S2).

### RMSD differences between protein variants and analysis of binding pockets

Structural superposition of the *Erg8*, *Erg9* and *HFA1* protein product variants was done using the SuperPose. The RMSD differences of alpha carbons, protein backbone, heavy atoms and overall RMSD between the variants of *Erg8*, *Erg9* and *HFA1* protein products are shown in Supporting Information S2. An overall RMSD of 2.02 Å was observed between the 3D-structures of the *Erg8* protein product from *S. cerevisiae* S288C and CEN.PK113-7D. No RMSD differences were observed between the *Erg9* protein product variants, indicating that the nsSNP acquired by CEN.PK113-7D has probably no effect on the 3D structure which is in line with the residue-residue interaction network analysis discussed above. The *HFA1* protein product variants showed an overall RMSD of 1.63 Å between their 3D-structures.

**Figure 3:**
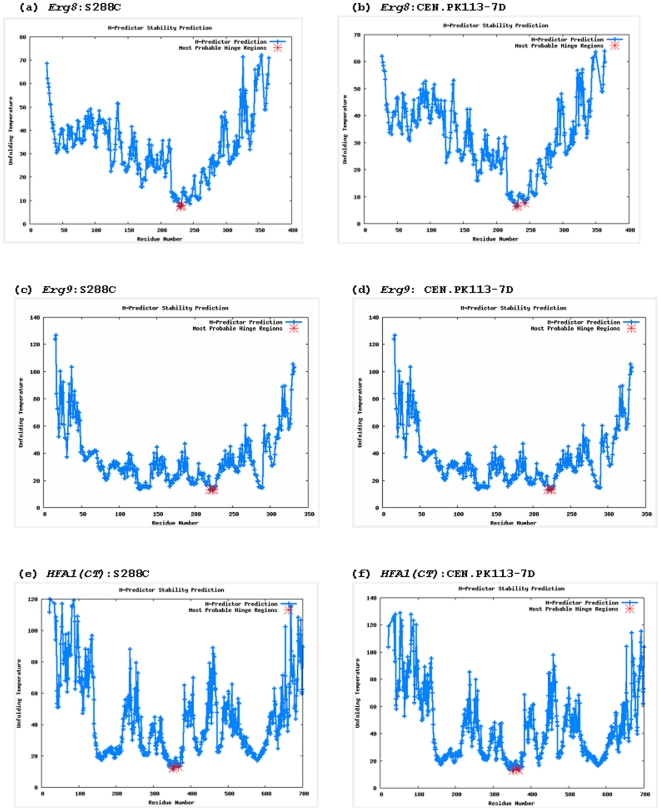
Identifying the hot-spot hinge regions of domain swapping in the variants of the *Erg8, Erg9* and *HFA1* protein products.

Analysis of the hinge regions using the H-predictor server also showed no differences in the case of the *Erg9* protein product variants, whereas a few differences were observed for the variants of *Erg8* and *HFA1* protein products (Figure 3). It should be noted that the predictions from the H-predictor server are not a measure of the protein's propensity for domain-swapping, but rather a structural propensity that a hinge region may result in domain swapping and also provide hint to the weakest regions that unfold prior to the compete unfolding of protein.

### Ligand binding sites of phosphomevalonate kinase, squalene synthase and carboxyl transferase domain

**Figure 4:**
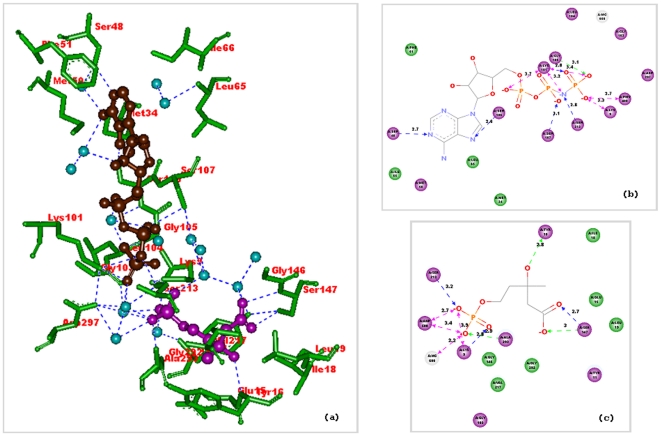
Ligand interaction diagrams for the active site of the phosphomevalonate kinase (PDB ID: 3GON). It was calculated that only 50% of the charge moieties of the ligands were in van der Waals contact with the protein. (a) The active site residues of phosphomevalonate kinase and their interaction with the ligands phosphomevalonate and AMPPNP through clusters of ordered water. Ligands are shown in ball and stick model. Phosphomevalonate is shown in brown color and AMPPNP in magenta color. Hydrogen bonds are shown as blue dashed lines. (b) Ligand binding pattern for AMPPNP and distance between the interacting amino acid residues calculated using the Accelry Discovery Studio version 2.5. (c) Ligand binding pattern for phosphomevalonate calculated and distance between the interacting amino acid residues using the Accelrys Discovery Studio version 2.5. The values shown are in Å units.

**Figure 5:**
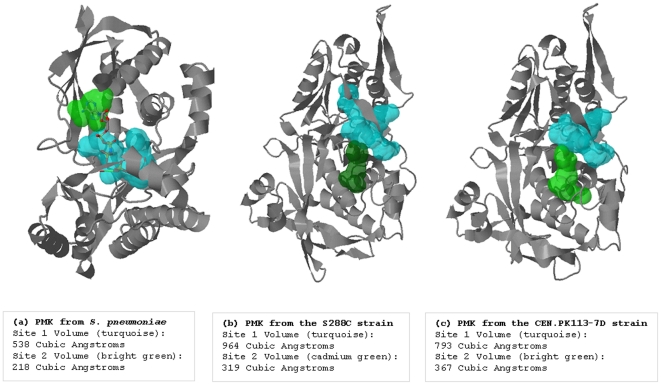
Ligand binding sites predicted using Q-SiteFinder. The two top ranked binding pockets were selected in each case. The differences between the phosphomevalonate kinase from *S. cerevisiae* S288C and CEN.PK113-7D strains can be clearly observed from the homology model structures shown. (a) Q-SiteFinder predictions for binding pockets in the phosphomevalonate kinase from *Streptococcus pneumoniae,* for which crystal structure data is available (PDB ID: 3GON). Q-SiteFinder was able to accurately predict the active site of 3GON with two binding pockets, one each for phosphomevalonate and AMPPNP. Both ligands are represented in stick model. (b) Q-SiteFinder predictions for binding pockets in phosphomevalonate kinase from *S. cerevisiae* S288C. (c) Q-SiteFinder predictions for binding pockets in phosphomevalonate kinase from *S. cerevisiae* CEN.PK113-7D.

Phosphomevalonate kinase catalyzes the phosphorylation of mevalonate-5-phosphate into mevalonate-5-pyrophosphate [34]. The template 3k17 (Lin0012 protein from *Listeria innocua*) that used for homology modeling of the *Erg8* protein products of *S. cerevisiae* S288C and CEN.PK113-7D showed high sequence and structural similarity with the crystal structure of phosphomevalonate kinase (3GON) from *Streptococcus pneumoniae*. The active site of the phosphomevalonate kinase (3GON) has been showed to have enough space to accommodate interconversion of the reactive and the nonreactive conformers [35]. The crystal structure of the ternary complex of phosphomevalonate kinase with phosphomevalonate and adenosine 5′-[β,γ-imido]triphosphate (AMPPNP) showed the presence of twenty-one ordered water molecules filling the interstices between the van der Waals surfaces of the phosphomevalonate kinase active site and its ligands. We analyzed the indirect binding pattern of amino acid residues with the ligands through clusters of ordered water molecules in the active site of phosphomevalonate kinase (Figure 4). A significant fraction of reactive regions is filled with a shell of water molecules, raising the issue about how phosphomevalonate kinase active site manages to prevent β,γ-bond hydrolysis during its catalytic cycle [35]. Analysis of the binding pockets in phosphomevalonate kinase from *S. cerevisiae* S288C and CEN.PK113-7D using Q-SiteFinder guided us to assume that nsSNPs acquired by CEN.PK113-7D strain were able to decrease the void space of the binding pocket which we consider as nature's engineering (Figure 5).

Squalene synthase is a membrane associated bifunctional enzyme that catalyzes the condensation of two molecules of farnesyl diphosphate (FPP) to give presqualene diphosphate (PSPP) and the subsequent rearrangement of PSPP to squalene [36]. In the human squalene synthase (PDB ID: 1ezf) it has been found that the five α helices surrounding the active center are structurally similar to that of other isoprenoid biosynthetic enzymes viz., farnesyl-diphosphate synthase, pentalenene synthase and 5-epi-aristolochene synthase [27]. When the crustal structures of these four enzymes were superimposed by Pandit *et al* [27], they all showed exactly the same orientation and interestingly less than 16% of the residues are identical in the superimposed parts, indicating that the pattern of the catalytic core is highly conserved structurally. It has also been suggested that all class-I isoprenoid enzymes may have evolved with similar structures regardless of the degree of amino acid sequence identity [37]. Based on these observations we expected that a single nsSNP coding for an amino acid substitution distant from catalytic core may have less structural impact on squalene synthase. Analysis of the binding pockets in homology models of squalene synthase from *S. cerevisiae* S288C and CEN.PK113-7D using Q-SiteFinder provided some evidence that the binding pockets of both *Erg9* protein products are similar (Supporting Information S3).

Biotin carboxylase domain catalyzes the ATP dependent carboxylation of a biotin group covalently linked to biotin carboxyl carrier protein, and then the carboxyl transferase domain catalyzes the transfer of the carboxyl group from biotin to acetyl-CoA to produce malonyl-CoA [28]. The amino acid sequences of carboxyl transferase domains have been found to be highly conserved among the eukaryotic multifunctional acetyl-CoA carboxylases and Zhang *et al* [38] have determined the crystal structure of the yeast carboxyl transferase domain in complex with CoA (PDB ID: 1OD2). Q-SiteFinder has been limited to PDB files with less than 10,000 atoms and was not able to predict the binding sites in the carboxyl transferase domain. So we used the CASTp server, an online tool that locates and measures pockets and voids on 3D protein structures [39]. However, CASTp was not able to correctly predict the location of the binding site in the carboxyl transferase domain where CoA molecule is known to bind [38]. We therefore superimposed the homology model structures of the *HFA1* protein products (CT domain) from *S. cerevisiae* S288C and CEN.PK113-7D using the Swiss-Pdb Viewer tool [40], to observe the structural effect of the nsSNP acquired by the CEN.PK113-7D strain (Supporting Information S3). We observed a few changes in the loops around the cavity where the CoA molecule is known to bind with the carboxyl transferase domain.

### Effects on ergosterol content and growth rate

#### Single over-expression constructs

**Figure 6:**
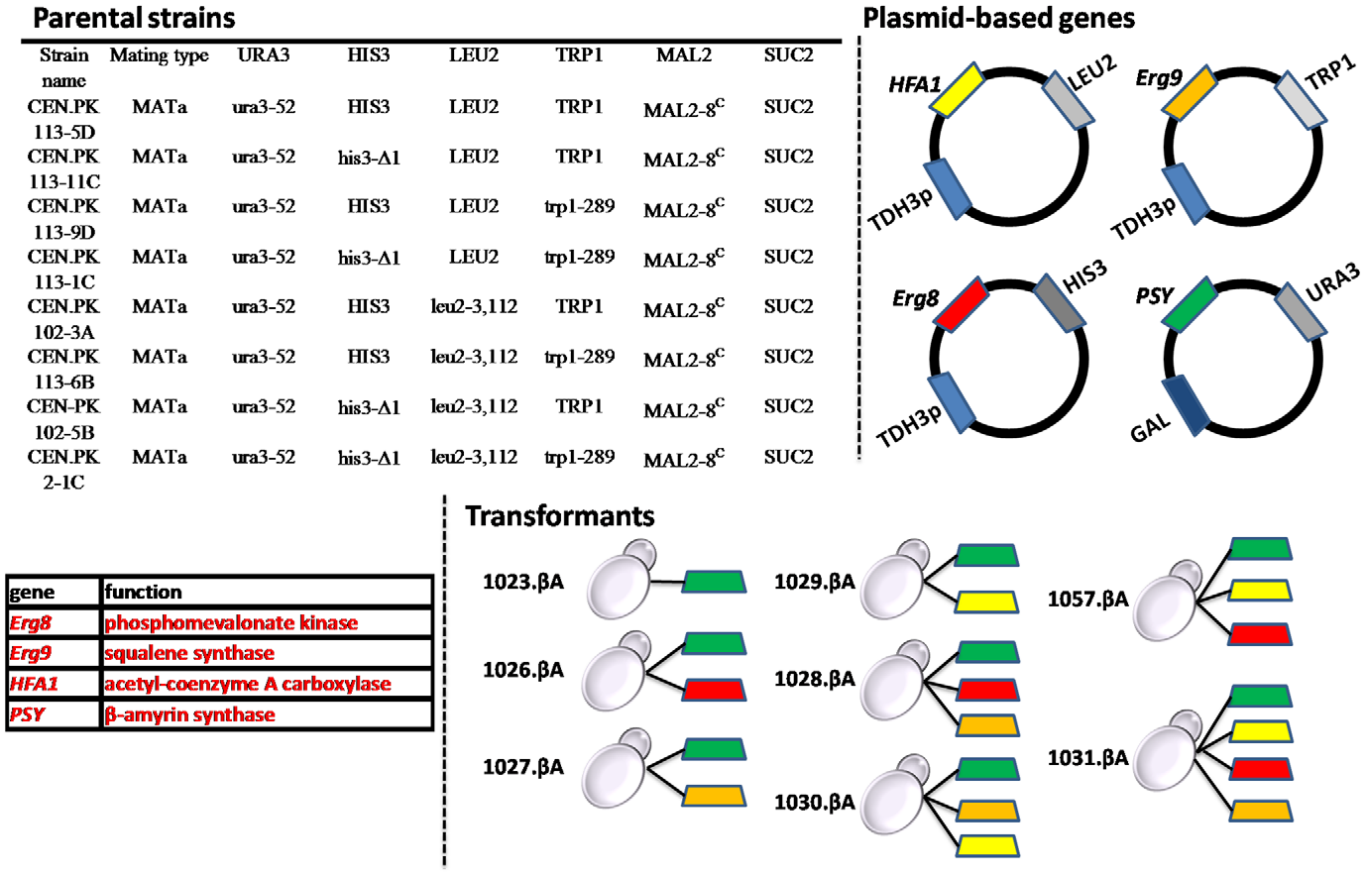
Systematic gene over-expression in CEN.PK strains harbouring a plasmid (pYES: GAL1 promoter/URA3 selection marker) with the PSY gene (*P. sativum*) coding for a β-amyrin synthase. The three genes *Erg8*, *Erg9*, and *HFA1* were ligated in different plasmids with the HIS3, TRP1 and LEU2 selection markers respectively, using the TDH3p promoter, and they were transformed in all combinations (single, double, triple over-expressions) to the respective parental strains leading to prototrophic strains. A visual representation of the final constructs containing from one (1) up to four (4) plasmids, as well as the name of the resulting strains, which is used in the text, is also given.

**Figure 7:**
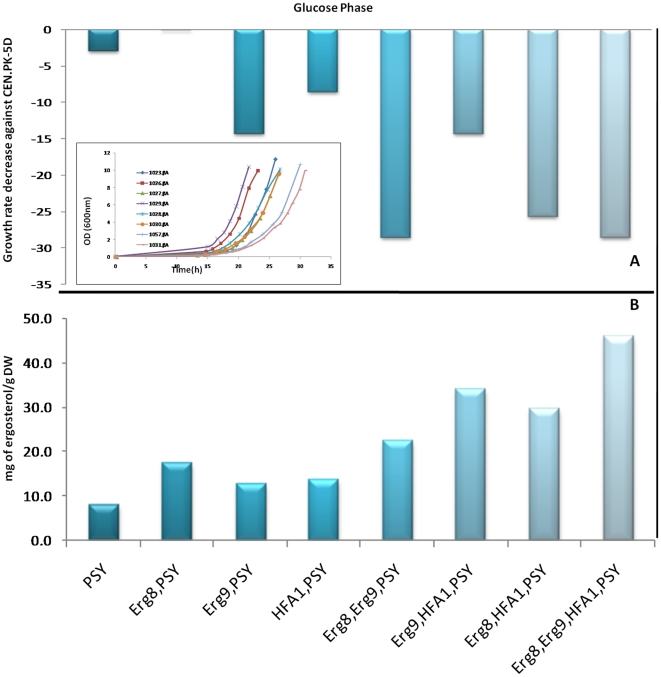
Physiological characterization of the reference and recombinant S. cerevisiae strains. (**A**) Bars represent the growth rates of the constructs relative to the reference strain when grown on glucose and the inset shows the growth curves. (**B**) Improved *in vivo* production of ergosterol from CEN.PK constructs. The production yields have been calculated at the end of the exponential growth. During this time period no β-amyrin was detected. *Strains: PSY (1023.βΑ), Erg8,PSY (1026.βΑ), Erg9,PSY (1027.βΑ), HFA1,PSY (1029.βΑ), Erg8,Erg9,PSY (1028.βΑ), Erg9,HFA1,PSY (1030.βΑ), Erg8,HFA1,PSY (1057.βΑ), Erg8,Erg9,HFA1,PSY (1031.βΑ).*

In order to test our hypothesis of a possible connection between the high levels of ergosterol in the CEN.PK strain and the proteins Erg8, Erg9 and HFA1, the corresponding three genes were over-expressed resulting to the strains 1026.βA, 1027.βA and 1029.βA, respectively (Figure 6). The three strains harbour also the PSY gene, however, the gene was under the control of the GAL1 promoter and no β-amyrin is produced during the glucose phase. This design allowed us to discern the effect of the over-expressions on the ergosterol content when there is no competition between the sterol pathway and the production of terpenes and on the second phase of the cultivation (ethanol consumption) to actually monitor the flux redirection among the two pathways for the available precursor (oxidosqualene). Indeed during our cultivations and until glucose was exhausted there was no β-amyrin production detected. On the other hand, the ergosterol content was significantly affected from the over-expressions when the constructs were compared with the control strain 1023.βA (Figure 7). The yield of ergosterol per gram of DW at the end of the exponential phase was ∼1.6-fold higher in the strains 1027.βA and 1029.βA compared to the 8.1 mg of ergosterol/g of DW that was determined for the control strain. In addition the specific growth rate of 1027.βA and 1029.βA was 14% and 9% lower than the reference strain (CEN.PK-5D), while the difference between the 1023.βA and the reference strain was negligible (3%). However, the most promising strain appeared to be 1026.βA, which reached an ergosterol content of 17.7 mg/g of DW, with no effect on the specific growth rate that remained unaltered compared to the reference strain.

#### Double and triple over-expression constructs.

Optimization of a secondary metabolite phenotype, such as ergosterol production, obviously depends on the modulation of several genes. With the intention to test a possible synergy in the resulting phenotype between the *Erg8*, *Erg9* and *HFA1* genes, the three double over-expression strains were also constructed. Figure 7 summarizes the results of the multiple gene over-expression constructs which seem to be of considerable interest. The three combinations resulted in the strains 1028.βA, 1030.βA and 1057.βA, which outperformed in ergosterol level the single over-expression constructs. The observed higher ergosterol levels of the double over-expression constructs it was not surprising and it could be predicted since the single over-expressions either enhanced (*Erg8*, *HFA1*) or matched (*Erg9*) the ergosterol level of the control strain. While a combination of *Erg8* and *Erg9* (1028.βA) had moderate effects on the ergosterol yield compared to the effect of the *Erg8* alone (1026.βA), this was not the case for the other two strains. The 1057.βA (*Erg8, HFA1)* strain reached an ergosterol level of 30 mg/g of DW, while even more impressive was the 34.3 mg of ergosterol/g of DW for the 1030.βA (*Erg9*, *HFA1*) strain. At the same time the specific growth rate of the 1030.βA appeared to be less affected by the over-expressions (∼14% decrease) compared to the other two strains which presented an >25% decrease on their growth rate (Figure 7). The presence of multiple plasmids within the yeast cell can be responsible for this profound impact on the cellular physiology since they often impose a metabolic burden on the cell. The highest ergosterol content was observed when the *Erg8*, *Erg9* and *HFA1* genes were simultaneously over-expressed. The quantification of the ergosterol content for the strain named 1031.βA revealed an amount as high as 46 mg/g DW, while the specific growth rate was not lower than the double constructs 1028.βA, and 1057.βA.

### β-amyrin production

#### Single over-expressions

**Figure 8:**
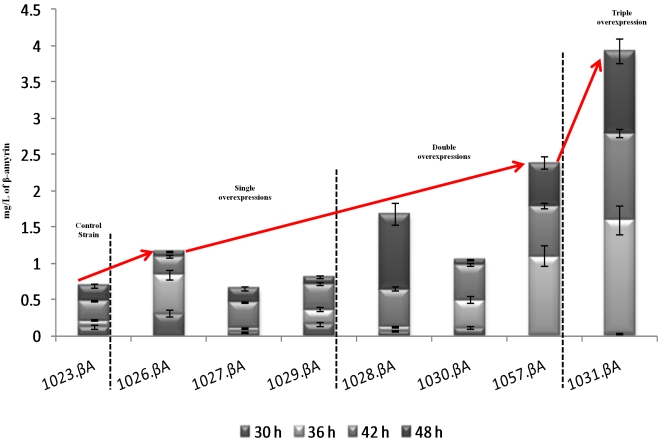
Production of β-amyrin is shown over 48 h for the constructs with single, double and triple over-expressions of *Erg8*, *Erg9,* and *HFA1,* and the control strain. The β-amyrin was detected after the exhaustion of glucose and the initiation of the consumption of ethanol that had produced by the strains. The data shown as total production are means for two independent cultivations for each strain.

In the second phase of the cultivation, and while all glucose had been consumed, the growth of the constructs was based on the ethanol consumption. During that period the production of β-amyrin was observed and the effect of the over-expression of *Erg8*, *Erg9*, and *HFA1* was evaluated. The strain 1023.βA was harbouring only the β-amyrin synthase gene and no other modification for higher expression was applied. Our metabolic engineering strategy was assessed based on the production level of this control strain. After a total cultivation time of 48 h the 1023.βA reached a maximum value of β-amyrin of 0.69 mg/L (Figure 8). In the strain 1027.βA, despite the fact that the over-expression of *Erg9* led to increased ergosterol yield in the glucose phase compared to the control strain, this positive effect was not reflected in the β-amyrin production levels during the ethanol phase (Figure 8). The final β-amyrin concentration was 0.66 mg/L while the ergosterol yield was 11 mg/g of DW, slightly lower than the 11.5 mg/g of DW of the control strain (data of ergosterol in the ethanol phase not shown). On the other hand over-expression of *HFA1* and *Erg8* did enhance the strains capacity to produce β-amyrin. As shown in Figure 8 after 48 h the strain 1029.βA produced 0.82 mg/L of β-amyrin. A further increase in the production levels was obtained from the strain 1026.βA with over 1.6-fold improvement compared to the control strain. The 1.17 mg/L of β-amyrin for 1026.βA was accompanied with an ergosterol yield of 12 mg/g of DW, higher than the control strain and the 1029.βA (8.9 mg of ergosterol/g of DW).

#### Double and triple over-expressions

We further monitor the β-amyrin changes triggered by the simultaneous up-regulation of the *Erg8*, *Erg9*, and *HFA1* genes in all the different combinations and the results are summarized in Figure 8. Surprisingly, even though *Erg9* over-expression had no marked impact on the β-amyrin measured, in combination with *Erg8* and *HFA1* over-expression the production was positively altered. The strain 1030.βA (*Erg9, HFA1*) produced 1.05 mg/L of β-amyrin, a 59% and 28% increase compared to the 1027.βA (*Erg9*) and 1029.βA (*HFA1*) respectively, having single over-expressions. The ergosterol yield of the 1030.βA was also rather high (25.7 mg/g of DW) but as in the case of the single over-expressions lower than the observed yield on glucose phase (34.3 mg/g of DW). On the other hand, the change in the β-amyrin observed for 1028.βA (*Erg8*, *Erg9*) was significantly greater than the 59% increase seen in the 1030.βA. The 1028.βA can accumulate up to 1.68 mg/L of β-amyrin, a 154% increase compared to the 1027.βA (*Erg9*) but only 43% increase compared to the 1026.βA (*Erg8*). The ergosterol yield for the strain 1028.βA was 31.3 mg/g of DW. However, from all the double over-expression constructs the combination of *Erg8* and *HFA1* (1057.βA) was the most attractive. The difference in the β-amyrin levels between the 1057.βA and the control strain appear to be over twice the difference than the best single over-expression achieved (1026.βA). The 2.39 mg/L of β-amyrin that 1057.βA produced were an increase of 246% compared to the control strain, while maintaining the high ergosterol levels (34 mg/g of DW). The construct with the triple over-expression of *Erg8*, *Erg9*, and *HFA1* outperformed the production of all the single and double over-expression constructs by a great extend. The final concentration of β-amyrin from the 1031.βA strain represents an increase of almost 500% compared to the control strain. The 3.93 mg/L of β-amyrin for the 1031.βA was a 3.4-times more β-amyrin than the best single over-expression construct (1026.βA) and 1.6-times more than the best double over-expression construct (1057.βA). At the same time the ergosterol content of the 1031.βA was higher than during the glucose phase and the highest observed compared to all the other strains (69.6 mg/g of DW).

## Discussion

Triterpenoids are a large class of isoprenoidal natural products present in higher plants. Among them, oleanane type triterpenes, which are produced from β-amyrin, are one of the most common triterpenes, along with ursane type triterpenes produced from α-amyrin. β-amyrin in particular serves as the olefin precursor to a wide range of downstream products. The action of oxidative enzymes and glycosyltransferases convert β-amyrin to various triterpene saponins. These saponins exhibit a wide range of both structural diversity and biological activity (antimicrobial, insecticidal agents) and therefore are regarded as important and promising sources for medicinal compounds. The effect of plant saponins on low-density lipoprotein cholesterol absorption and arterial atherosclerosis has received much attention, leading to the development of several cholesterol-reducing dietary supplements [41]. The formation of these complex carbon skeletons through a series of protonation, cyclization, rearrangement and deprotonation reactions of 2,3-oxidosqualene is well documented in the famous biogenetic isoprene rule [42]. Although triterpene synthases have been expressed in microbial hosts such as *S. cerevisiae* there has been little effort made so far to engineer the metabolism of a microbial host for enhanced production of triterpenes. Imbalances in gene expression can lead to –over or –under production of enzymes in the pathway, accumulation of toxic metabolic intermediates, and metabolic burden on the host, all of which result in suboptimal product titers [43]. A novel metabolic engineering strategy for designing a triterpenoid-yeast-production-platform is presented here based on the whole genome sequencing of *S. cerevisiae* CEN.PK recently completed by Otero *et al* [12].

The non-synonymous SNPs, the so called non-silent SNPs, which are single nucleotide variations in the coding regions that gives ‘birth’ to amino acid mutations, are often involved in the modulation of protein function. Understanding the effect of individual amino acid mutations on a protein/enzyme function or stability is useful for altering its properties for wide variety of engineering studies. Since measuring the effects of mutations experimentally is a laborious process, a variety of computational methods and algorithms have been devised to predict these effects *in silico* [44]-[50]. Bioinformatics approaches to predict the effect of mutations on protein stability utilizes the sequence alignment information of evolutionarily related sequences [51] or protein families or rely on physicochemical modeling of the mutation augmented by information obtained from statistical analyses of protein sequences and three-dimensional structures [52]. Computational approaches for predicting the effect of amino acid mutations has proven to be surprisingly successful, with a wide range of studies supporting them [53]-[56]. Different computational algorithms provide valuable insights to explore relationships between beneficial mutations and phenotypic variation and speed up both fundamental and industrial applied research [57]. *Erg8*, *Erg9*, and *HFA1* genes are part of the sterol and fatty acid biosynthesis in *S. cerevisiae. S. cerevisiae* CEN.PK contains an unusually high content of ergosterol and fatty acids compared to other *S. cerevisiae* strains [11]. When Otero and colleagues [12] compared the genome-wide sequence of CEN.PK with S288C they identified a number of SNPs in these 3 genes. Our hypothesis in this study was that these SNPs are linked to the observed phenotype in CEN.PK, by the formation of more efficient Erg8, Erg9 and HFA1 proteins, influencing the flux towards the two pathways. Our hypothesis was supported by the use of an array of computational tools that there is a positive effect of the nsSNPs on the protein structure-stability-function of the Erg8, Erg9 and HFA1.

The *Erg8* codes for a phosphomevalonate kinase, an essential cytosolic enzyme which catalyzes the reaction ATP+(*R*)-5-phosphomevalonate = ADP+(*R*)-5-diphosphomevalonate. An indirect over-expression of *Erg8* through an enhanced activity of UPC2 (a global transcription factor regulating the biosynthesis of sterols in *S. cerevisiae*) for terpenes production has been studied by Ro *et al* [58]. However, UPC2 as a single modification had only a modest effect on amorphadiene production. A negative effect of the enhanced UPC2 activity on the epicedrol production, a sesquiterpene originating from FPP, was observed by Jackson *et al* [59]. However, in the present study the direct over-expression of *Erg8* resulted in higher ergosterol content than the control strain during growth on glucose, which was then reflected in the ethanol phase in the 1.6-fold higher production of β-amyrin compared to the control strain.

The *Erg9* codes for a squalene synthase that joins two farnesyl pyrophosphate moieties in the reaction 2 farnesyl diphosphate = diphosphate+presqualene diphosphate. There have been several studies targeting *Erg9* as an attempt to increase precursor availability for terpenes production. In the case of Shimada *et al* [60] disruption of the *Erg9* gene as a single modification in *Candida utilis* had no significant effect on lycopene production. On the other hand Paradise *et al* [61] increased by 5-fold the production of amorphadiene by down-regulating the *Erg9*, however this improvement was in a strain background with several other genetic modifications. In line with the above two studies were the effects of *Erg9* over-expression in the β-amyrin production observed here. While *Erg9* over-expression as a single metabolic engineering strategy had no positive effect on β-amyrin production, in combination with *Erg8* over-expression there was a 2.4-fold improvement compared to the control strain.

The HFA1 is a mitochondrial acetyl-coenzyme A carboxylase that catalyzes the production of malonyl-CoA in fatty acid biosynthesis through the reaction ATP+acetyl-CoA+HCO_3_^−^ = ADP+phosphate+malonyl-CoA. Interestingly, by enhancing the expression level of HFA1 the production level of β-amyrin was improved by 1.2 times. Kizer *et al* [62] engineered an *E. coli* strain to produce high levels of terpenoids, however, further optimization led to an imbalance in carbon flux and the accumulation of the pathway intermediate 3-hydroxy-3-methylglutaryl-coenzyme A (HMG-CoA), which proved to be toxic to *E. coli.* Their results indicated that HMG-CoA inhibits fatty acid biosynthesis in the microbial host, leading to generalized membrane stress. The cytotoxic effects of HMG-CoA accumulation could be counteracted by the addition of palmitic acid and oleic acid, and it is possible that the positive effect of *HFA1* over-expression in ergosterol and β-amyrin levels that we observed in our study to be a mechanism of the cell to deal with the high HMG-CoA concentrations. Over-expression of *HFA1* with concomitant over-expression of *Erg8* led to the highest production of β-amyrin in between all the single and double over-expression constructs, while the final concentration was 3.5-fold higher than the control strain. Further improvement in the β-amyrin production level was achieved by the triple over-expression construct.

In summary we have created a strain of *S. cerevisiae* capable of producing 500% more β-amyrin than the control strain by the simultaneous over-expression of *Erg8*, *Erg9* and *HFA1*. To the best of our knowledge the only metabolic engineering work applied for β-amyrin production has been performed by Kirby *et al* [63]. By manipulating the two key enzymes in the pathway, HMG-CoA reductase and lanosterol synthase, Kirby and colleagues improved the β-amyrin production by 50%. This was a 10-fold lower improvement than the one achieved through our metabolic engineering strategy. However, in the study of Kirby *et al* the final titer of β-amyrin was 6 mg/L [63].

In addition to the above modifications, a careful inspection of the metabolic pathways that include the acetyl-CoA molecule for SNPs could reveal more targets for redirecting the fluxes towards the mevalonate pathway. The supply of acetyl-CoA has been shown as an important parameter for the production of many secondary metabolites and in particular terpenoid molecules, as Shiba *et al* [64] demonstrated in their study.

However, it is important also to stress out that despite the very encouraging results from integrating protein computational analysis with metabolic engineering, there is a clear need for further experimental verification of our hypothesis. In order to increase our confidence that the SNPs in the three proteins are responsible for the differences observed in the ergosterol level between the strains, we should create point mutations in the CEN.PK genes to construct the respective version of the S288 strain and examine if the phenotype of S288 is restored in the CEN.PK and vice versa. This could potentially demonstrate the role of the SNPs in a flux level. Additionally, an isolation of the different versions of the S288 and CEN.PK proteins and the evaluation of their *in vitro* activity against their natural substrates would definitely strengthen the computational predictions regarding the beneficial effects caused by the SNPs in the proteins of CEN.PK. It would also be of interest to overexpress *Erg8*, *Erg9* and HFA1 in S288 and compare the obtained levels of β-amyrin in S288 and CEN.PK which may point out other limitations in creating a yeast β-amyrin hyper-producer.

In this work we propose that high-throughput genome sequencing of *S. cerevisiae* may serve as a commonplace tool, complimentary to transcriptomics and physiological characterization, to extract direct genotype to phenotype information. The analysis presented here serves as a foundation for comparative metabolic engineering SNP analysis, where in the future reference strains may be compared to their metabolically engineered derivatives that use directed evolution in order to answer what changes have made a strain a preferred microbial cell factory. Future work must also expand to the SNP analysis presented in the paper of Otero *et al* [12] to include all 13,787 SNPs, realizing phenotypic observations may not necessarily be linked directly to metabolic SNPs, but rather SNPs affecting larger regulatory mechanisms and networks, such as those governed by transcription factors.

## Materials and Methods

### Analysis of nsSNPs by Sequence Homology Based method

We used SIFT, a sequence homology based tool that **S**orts **I**ntolerant **F**rom **T**olerant amino acid substitutions, to find out the effect of nsSNPs in *Erg8*, *Erg9* and *HFA1* protein products. The SIFT algorithm relies solely on sequence to predict whether an amino acid substitution at a particular position in a protein will have a phenotypic effect. To predict the effect of an amino acid substitution, SIFT considers the information about the position at which the change occurred and the type of amino acid change. SIFT is a multistep procedure that, for a query sequence, (1) searches for similar sequences, (2) chooses closely related sequences that may share similar function, (3) obtain multiple alignment of these chosen sequences, and (4) calculates normalized probabilities for all possible substitutions at each position from the alignment. Substitutions at each position with normalized probabilities less than the chosen SIFT cutoff are predicted to be deleterious and those that are greater than or equal to the SIFT cutoff are predicted to be tolerated. Therefore, the accuracy for predicting the phenotype that results from an amino acid substitution based on sequence alignment of protein family members has been assumed to be better than using a generalized substitution scoring matrix [65].

### Homology modeling and Structure validation

Homology modeling of *Erg8*, *Erg9* and *HFA1* protein products was carried out using the Protein Model Portal (PMP) that provides a single interface to access 12.7 million comparative protein models across various protein structure databases (Release date: 2010/03/19) and also provides interactive services for template selection, target template alignment, model building and quality assessment [66]. PMP is a module of the Protein Structure Initiative Knowledge Base (PSI KB) developed by the Protein Structure Bioinformatics group at the SIB - Swiss Institute of Bioinformatics and the Biozentrum - University of Basel. The overall model quality of structures obtained from homology modeling were validated using ProSA-web Protein Structure Analysis tool [67]. ProSA-web calculates the overall quality *z*-score for a specific input structure and relates to the scores computed from all experimental structures deposited in Protein Data Bank (PDB). The *z*-score is displayed on a plot, so that low-resolution structures and approximate models obtained through homology modeling can be evaluated and compared against high resolution structures.

### Simulations for functional change in coding nsSNPs based on 3D structures

Structural analysis was performed for evaluating the structural stability of homology models for *Erg8*, *Erg9* and *HFA1* protein products from both *S. cerevisiae* S288C and CEN.PK113-7D strains. A measure of protein stability is the difference between the free energies of the folded and infolded states. We used Eris, a protein stability prediction server [68] that employs improved Medusa force field [69] for estimation of change in free energy difference (ΔΔ*G*) upon mutation. Eris features an all-atom force filed, a fast side-chain packing algorithm, and a backbone relaxation method for accurate protein stability predictions. To obtain information about *Accessible Surface Area (ASA)* changes caused by nsSNPs on protein structures, homology models of *Erg8*, *Erg9* and *HFA1* protein products from both *S. cerevisiae* S288C and CEN.PK113-7D were submitted to InterProPatch server [70] that shows surface region differences.

### Analysis of changes in Residue-Residue Interactions caused by individual nsSNPs

Analysis of changes in residue-residue interactions caused by nsSNPs on *Erg8*, *Erg9* and *HFA1* protein products was done at University of Cambridge -UK, using *Bongo* server (**B**onds **ON** **G**raph). *Bongo* uses graph theoretic measures to annotate nsSNPs and represent residue-residue interaction networks within proteins on graphs. A single amino acid substitution encoded by a nsSNP may often not only give rise to rearrangement of amino acid side chains near the mutation site, but also to a substantial local or global movement of polypeptide backbone. A major advantage of *Bongo* is that it considers the long-distance structural impact of a point mutation.

### Structural analysis and Scanning of binding pockets

To analyze the overall structural differences between the *Erg8*, *Erg9* and *HFA1* protein products of *S. cerevisiae* S288C and CEN.PK113-7D, we used SuperPose, a sophisticated structural superposition program that uniquely combines sequence alignment and difference distance (DD) matrix calculations to constrain its quaternion superposition algorithm [71]. Through H-Predictor server, we also analyzed putative hinge regions that are involved in protein oligomerization via the domain-swapping mechanism [72]. Using a simple contact-based potential for enthalpy and graph theory- based estimation for entropy, H-Predictor quantifies for each residue the propensity as the hinge region. Finally, the binding pockets of *Erg8*, *Erg9* and *HFA1* protein products from both *S. cerevisiae* S288C and CEN.PK113-7D were scanned using Q-SiteFinder [73] to find out the protein-ligand binding site differences caused by coding nsSNPs. The special feature of Q-SiteFinder is that it uses interaction energy and a simple van der Walls probe to locate energetically favourable binding sites. By scanning binding pockets, not only the ligand binding sites of a given protein can be identified, but also protein residues within a suitable range of the binding pocket are identified, which could be used for analysis of functional sites and comparison.

### Strains and Media

The strains used in this study as well as the construction process are shown in Figure 6. The plasmids (2micron multi-copy vectors) pPK529 (*Erg8*), pPK532 (*Erg9*) and pPK534 (*HFA1*) were transformed as single, double (in all combinations), and triple over-expressions to the respective parental strains. The genes were cloned between the TDH3 promoter and terminator region. In addition, a gene (PSY) coding for a β-amyrin synthase from the plant *Pisum sativum* (pea) [74] was transformed to the above CEN.PK over-expression mutants and the reference strain (CEN.PK-5D) using the commercially available pYES plasmid (Invitrogen) as described previously [75]. The final strains harbouring different combinations of plasmids and their designated names are shown in Figure 6. All the resulting strains were prototrophic. Cultures were maintained by plating in SCD medium and these stocks were used to inoculate the pre-cultures. Pre-cultures were grown in shake flask cultures on defined mineral medium [76], supplemented with vitamins, adjusted to pH 6.0 and containing 2% (w/v) glucose.

### Batch Cultivation Conditions

To determine the physiological characteristics of the different yeast strains they were grown in batch cultivations in well-controlled 2 L bioreactors with a working volume of 1.5 L. In brief, the cultures were fed with a defined mineral medium as described above, containing glucose (2% w/v) as the limited nutrient. The bioreactors were equipped with two disc-turbine impellers rotating at 600 rpm. The pH was kept constant at 5.0 by addition of 2 M KOH or HCl and the temperature was maintained at 30 °C. Air was used for sparging the bioreactor at a constant flow rate of 1.0 vvm (volume of gas per volume of liquid per minute).

### Analysis of substrates and products

Cell dry weight was determined using nitrocellulose filters (pore size 0.45 µm, Gelman Sciences). Fermentation samples were immediately filtered and stored at −20 °C until analysis. The concentrations of glucose, ethanol, glycerol, acetate, succinate, and pyruvate were determined by HPLC as described previously [77].

### Sampling, extraction, determination and analysis of ergosterol and β-amyrin

For the analysis of ergosterol and β-amyrin dublicate biological samples were collected (30 ml), centrifuged at 4000 rpm for 3 min and the pellets immediately stored at −20 °C. The defrosted pellet was re-suspended in 2 ml 20% w/v sodium hydroxide in 50% ethanol. The mixture was transferred to glass tube and was kept in boiling water for 5 min with occasional shaking. Subsequently, 1 ml of 20% w/v sodium hydroxide in 50% ethanol and 2 ml hexane were added, followed by vortex-mixing for 30–60 seconds. The tubes were centrifuged for 5 min at 1000 rpm and the hexane phase was extracted for further analysis. After drying the samples were derivatised by adding 50 µl BSTFA and 50 µl pyridine [78], dried and dissolved in 75 µl toluene.

GC-MS was used for quantifying the ergosterol and β-amyrin content of the samples. The injection volume was 1 µl in a Rtx-5 ms (30 meters, 0.25 mm ID) column with helium carrier. The column temperature was maintained at 240 °C for 2 min, elevated to (10 degrees/min) 330 °C and then held for 6.5 min at 330 °C. Authentic β-amyrin and ergosterol were derivatized and analyzed in GC-MS in the same manner.

## Supporting Information

Supporting Information S1 SIFT predictions for the effect of amino acid substitutions caused by nsSNPs along Erg8, Erg9 and HFA1 protein products. Homology modeling and structure validation of Erg8, Erg9, and HFA1 protein products
(DOCX)

Supporting Information S2 Protein stability calculations for Erg8, Erg9 and HFA1 protein products of both S.cerevisiae S288C and S.cerevisiae CEN.PK113-7D strains. Accessible Surface Area (ASA) calculations for Erg8, Erg9 and HFA1 protein products of both S.cerevisiae S288C and S.cerevisiae CEN.PK113-7D RMSD differences between Erg8, Erg9 and HFA1 protein product variants of S.cerevisiae S288C and S.cerevisiae CEN.PK113-7D strains Graph theoretic measures of the structural effects in proteins caused by individual nsSNPs
(DOCX)

Supporting Information S3 Ligand binding sites of the squalene synthase predicted using the Q-SiteFinder and structural analysis of the carboxyl transferase domain.
(PDF)
