# Supplementary material for: Linking Genotype and Phenotype of Saccharomyces cerevisiae Strains Reveals Metabolic Engineering Targets and Leads to Triterpene Hyper-Producers
Source: PLoS One. 2011 Mar 18;6(3):e14763. doi: 10.1371/journal.pone.0014763 (PMC3060802; doi:10.1371/journal.pone.0014763)
Supplement: Supporting Information S1 — SIFT predictions for the effect of amino acid substitutions caused by nsSNPs along Erg8, Erg9 and HFA1 protein products. Homology modeling and structure validation of Erg8, Erg9, and HFA1 protein products (DOCX) [file pone.0014763.s001.docx]

**Tables 1 (a) to (f):** SIFT predictions for the effect of amino acid substitutions caused by nsSNPs along Erg8, Erg9 and HFA1 protein products.

| Amino acid position | R49 | R75 | R192 | R247  **(a)** SIFT predictions considering *S. cerevisiae* S288C as wild strain and *S. cerevisiae* CEN.PK113-7D as mutant strain that acquired respective amino acid substitutions along *Erg8* protein product. |
| --- | --- | --- | --- | --- |
| Erg8:S288C | G | S | A | D |
|  | \|  \| \| --- \| | \|  \| \| --- \| | \|  \| \| --- \| | \|  \| \| --- \| |
| Erg8:CEN.PK113-7D | E | T | S | N |
| SIFT score | 1 | 0.25 | 1 | 0.22 |
| SIFT result | Tolerant | Tolerant | Tolerant | Tolerant |

| Amino acid position | R49 | R75 | R192 | R247  **(b)** SIFT predictions considering *S. cerevisiae* CEN.PK113-7D as wild strain and *S. cerevisiae* S288C as mutant strain that acquired respective amino acid substitutions along *Erg8* protein product. |
| --- | --- | --- | --- | --- |
| Erg8:CEN.PK113-7D | E | T | S | N |
|  | \|  \| \| --- \| | \|  \| \| --- \| | \|  \| \| --- \| | \|  \| \| --- \| |
| Erg8:S288C | G | S | A | D |
| SIFT score | 0.11 | 0.81 | 0 | 0.72 |
| SIFT result | Borderline | Tolerant | Intolerant | Tolerant |

**(c)** SIFT predictions considering *S. cerevisiae* S288C as wild strain and *S. cerevisiae* CEN.PK113-7D as mutant strain that acquired respective amino acid substitutions along *Erg9* protein product.

| Amino acid position | 286 |
| --- | --- |
| Erg9:S288C | G |
|  | \|  \| \| --- \| |
| Erg9:CEN.PK113-7D | S |
| SIFT score | 0.8 |
| SIFT result | Tolerant |

**(d)** SIFT predictions considering *S. cerevisiae* CEN.PK113-7D as wild strain and *S. cerevisiae* S288C as mutant strain that acquired respective amino acid substitutions along *Erg9* protein product.

| Amino acid position | R286 |
| --- | --- |
| Erg9:CEN.PK113-7D | S |
|  | \|  \| \| --- \| |
|  |  |
| Erg9:S288C | G |
| SIFT score | 0.81 |
| SIFT result | Tolerant |

**(e)** SIFT predictions considering *S. cerevisiae* S288C as wild strain and *S. cerevisiae* CEN.PK113-7D as mutant strain that acquired respective amino acid substitutions along *HFA1* protein product.

| Amino acid position | 579 | 877 | 971 | 1056 | 1273 | 1798 |
| --- | --- | --- | --- | --- | --- | --- |
| HFA1:S288C | S | K | E | A | I | I |
|  | \|  \| \| --- \| | \|  \| \| --- \| | \|  \| \| --- \| | \|  \| \| --- \| | \|  \| \| --- \| | \|  \| \| --- \| |
| HFA1:CEN.PK113-7D | G | E | K | T | T | T |
| SIFT score | 0.01 | 0.28 | 0.04 | 0.86 | 0.85 | 0.34 |
| SIFT result | Intolerant | Tolerant | Intolerant | Tolerant | Tolerant | Tolerant |

**(f)** SIFT predictions considering *S. cerevisiae* CEN.PK113-7D as wild strain and *S. cerevisiae* S288C as mutant strain that acquired respective amino acid substitutions along *HFA1* protein product.

| Amino acid position | 579 | 877 | 971 | 1056 | 1273 | 1798 |
| --- | --- | --- | --- | --- | --- | --- |
| HFA1:CEN.PK113-7D | G | E | K | T | T | T |
|  | \|  \| \| --- \| | \|  \| \| --- \| | \|  \| \| --- \| | \|  \| \| --- \| | \|  \| \| --- \| | \|  \| \| --- \| |
| HFA1:S288C | S | K | E | A | I | I |
| SIFT score | 0.01 | 1 | 0.03 | 0.5 | 0.08 | 0.45 |
| SIFT result | Intolerant | Tolerant | Intolerant | Tolerant | Potentially Intolerant | Tolerant |

*Homology modeling and structure validation of Erg8, Erg9 and HFA1 protein products*

*Erg8 protein product - Phosphomevalonate Kinase:*

- The crystal structure of Lin0012 protein (PDB ID: 3k17) comprises of four chains. Secondary structure alignments for amino acid sequences of the respective 3k17 chain against the amino acid sequence of *Erg8* protein product resulted into selection of 3k17C as the best template for homology modeling.
- The FATCAT (**F**lexible structure **A**lignmen**T** by **C**haining **A**ligned fragment pairs with **T**wists) algorithm optimizes structural alignment by minimizing the number of rigid-body movements (twists) around pivot points (hinges) introduced in the reference protein and therefore achieves more accurate structure alignments.

*Erg9 protein product – Squalene Synthase:*

- The crystal structure of the human squalene synthase (PDB ID: 1ezf) comprises of three chains (A, B and C chains). Secondary structure alignments of the amino acid sequences respective 1ezf chain against amino acid sequence of the *Erg9* protein product resulted into selection of 1ezfC as the best template for homology modeling.

**Table 2:** Scores generated using ClustalW for secondary structure alignment of *Erg8* protein product against 3k17 A, B, C and D chains.

| SeqA | Name | SeqB | Name | Alignment Score |
| --- | --- | --- | --- | --- |
| 1 | Erg8 | 2 | 3k17C | 87 |
| 1 | Erg8 | 3 | 3k17D | 86 |
| 1 | Erg8 | 4 | 3k17A | 87 |
| 1 | Erg8 | 5 | 3k17B | 86 |
| 2 | 3k17C | 3 | 3k17D | 96 |
| 2 | 3k17C | 4 | 3k17A | 98 |
| 2 | 3k17C | 5 | 3k17B | 98 |
| 3 | 3k17D | 4 | 3k17A | 97 |
| 3 | 3k17D | 5 | 3k17B | 96 |
| 4 | 3k17A | 5 | 3k17B | 99 |


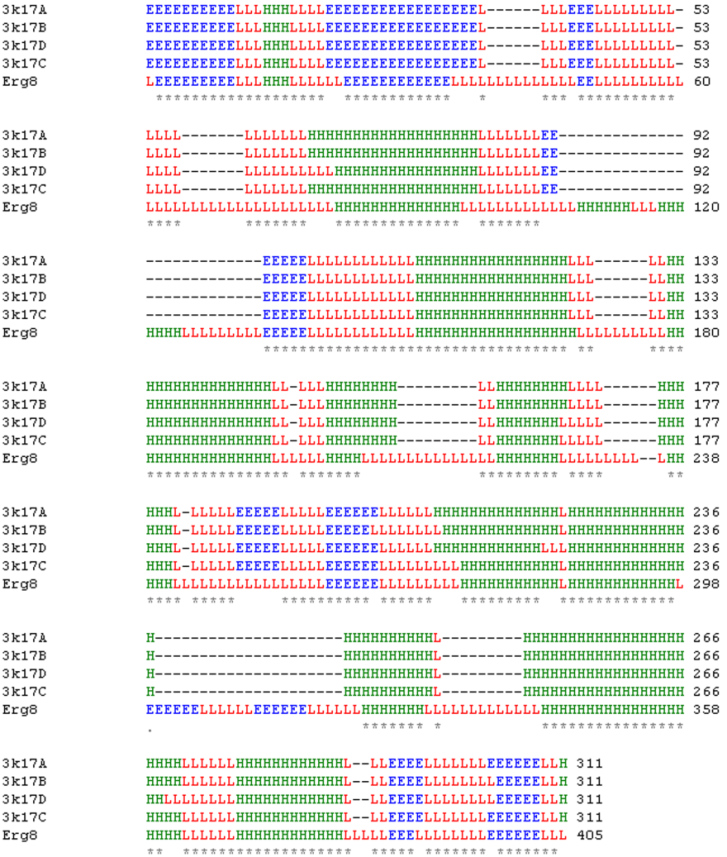


**Figure 1(a):** Secondary structure alignment of *Erg8* protein product with 3k17 A, B, C and D chains.


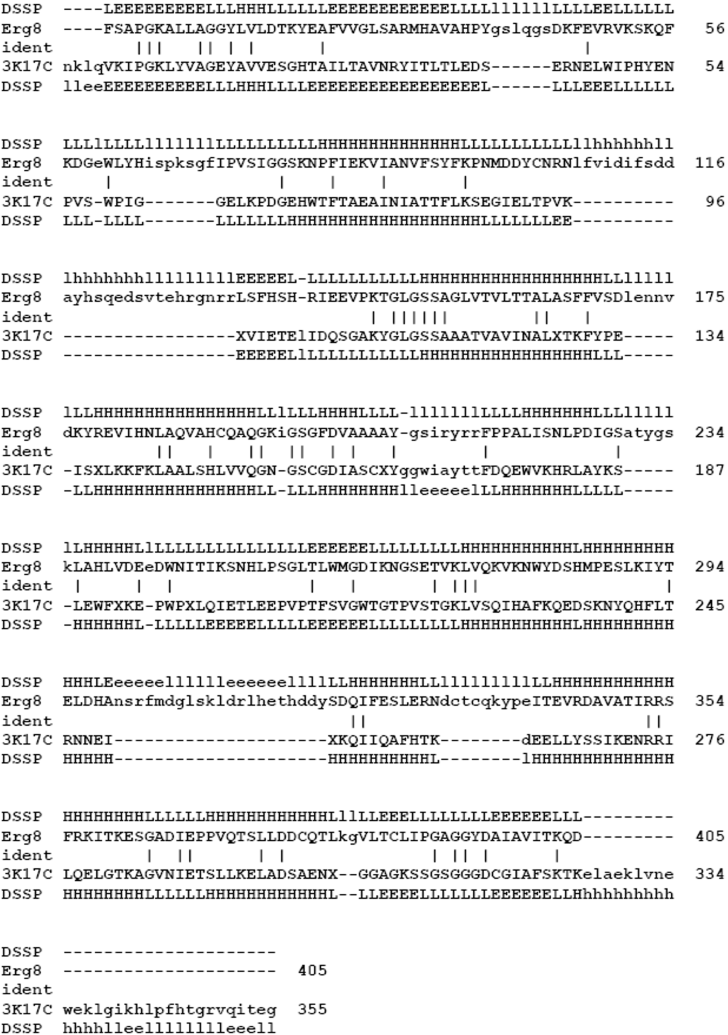


**Figure 1(b):** Pairwise structural alignment of 3k17C and *Erg8* protein product. Dali server uses DSSP program (Carter *et al*., 2003) for secondary structure assignments from three-dimensional co-ordinates of the given protein structure. Secondary structure Notation: three-state secondary structure definitions by DSSP (H=helix, E=sheet, L=coil) are shown above the amino acid sequence. Structurally equivalent residues are in uppercase, structurally non-equivalent residues (e.g. in loops) are in lowercase. Amino acid identities are marked by vertical bars.

Table 3: 3K17.A (chain 1) vs. representatives of other sequence clusters (chain 2)

| Chain 1 | Chain 2 | Title | P-value | Score | Rmsd | Len1 | Len2 | %Sim1 | %Sim2 |
| --- | --- | --- | --- | --- | --- | --- | --- | --- | --- |
| 3K17.A | 3GON.A | Phosphomevalonate kinase | 0 | 820.59 | 1.65 | 355 | 329 | 92 | 99 |
| 3K17.A | 2DEJ.A | Probable galactokinase | 1.17E-14 | 652.79 | 2.51 | 355 | 346 | 87 | 89 |
| 3K17.A | 1PIE.A | Galactokinase | 1.26E-13 | 644.54 | 2.58 | 355 | 388 | 90 | 82 |
| 3K17.A | 2HFS.B | Mevalonate kinase, putative | 1.49E-13 | 662.36 | 3.03 | 355 | 326 | 82 | 90 |
| 3K17.A | 1WUU.A | Galactokinase | 1.61E-12 | 607.97 | 2.66 | 355 | 391 | 89 | 81 |
| 3K17.A | 1KKH.A | Mevalonate Kinase | 2.52E-12 | 574.64 | 2.97 | 355 | 317 | 86 | 97 |
| 3K17.A | 3K85.A | D-glycero-D-manno-heptose 1-phosphate kinase | 2.92E-12 | 536.22 | 2.52 | 355 | 306 | 80 | 93 |
| 3K17.A | 1KVK.A | mevalonate kinase | 4.33E-12 | 644.53 | 3.28 | 355 | 378 | 87 | 82 |
| 3K17.A | 2OI2.A | Mevalonate kinase | 5.43E-12 | 577.51 | 3.12 | 355 | 285 | 77 | 95 |
| 3K17.A | 1H72.C | Homoserine Kinase | 7.64E-11 | 503.15 | 3.01 | 355 | 296 | 83 | 99 |
| 3K17.A | 2A2C.A | N-acetylgalactosamine kinase | 4.70E-10 | 559.9 | 3.07 | 355 | 446 | 90 | 72 |
| 3K17.A | 2HK2.B | Diphosphomevalonate decarboxylase | 5.07E-10 | 498.76 | 3.12 | 355 | 331 | 83 | 89 |
| 3K17.A | 2GS8.A | mevalonate pyrophosphate decarboxylase | 1.50E-09 | 467.95 | 3.04 | 355 | 316 | 80 | 90 |
| 3K17.A | 3HUL.B | Homoserine kinase | 1.57E-09 | 428.37 | 2.77 | 355 | 269 | 74 | 98 |
| 3K17.A | 3LTO.B | Mevalonate diphosphate decarboxylase | 3.55E-09 | 467.55 | 3.03 | 355 | 314 | 73 | 83 |

**P-value** - The P-value of this alignment (from FATCAT).

**Title -** Name of respective Chain 2 protein

**Score** - The raw alignment score (from FATCAT).

**RMSD** - The RMSD value of the alignment.

**Len1** - The length of the chain 1.

**Len2** - The length of the chain 2.

**%Sim1** - The % of residues in chain 1 that are aligned.

**%Sim2** - The % of residues in chain 2 that are aligned.

**(a)** **(b)**


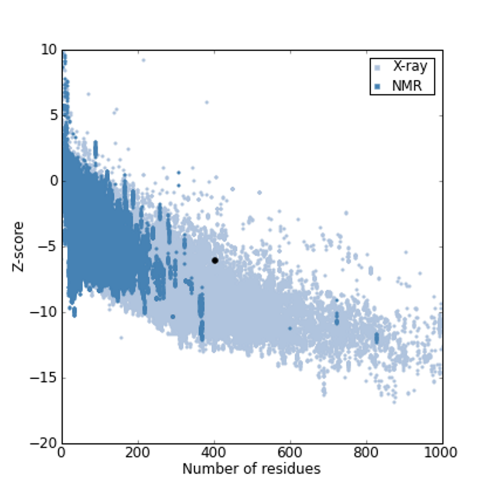

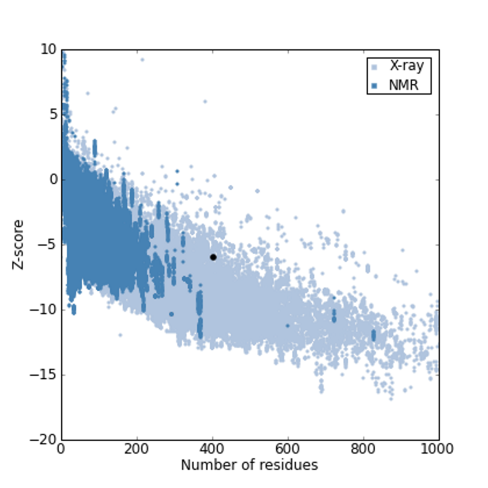


**Figure 2:** Quality assessment of homology models by ProSA-web. (**a)** *Erg8* protein product from S288C. *Z*-Score: -6.1

**(b)** *Erg8* protein product from CEN.PK113-7D. *Z*-Score: -5.97


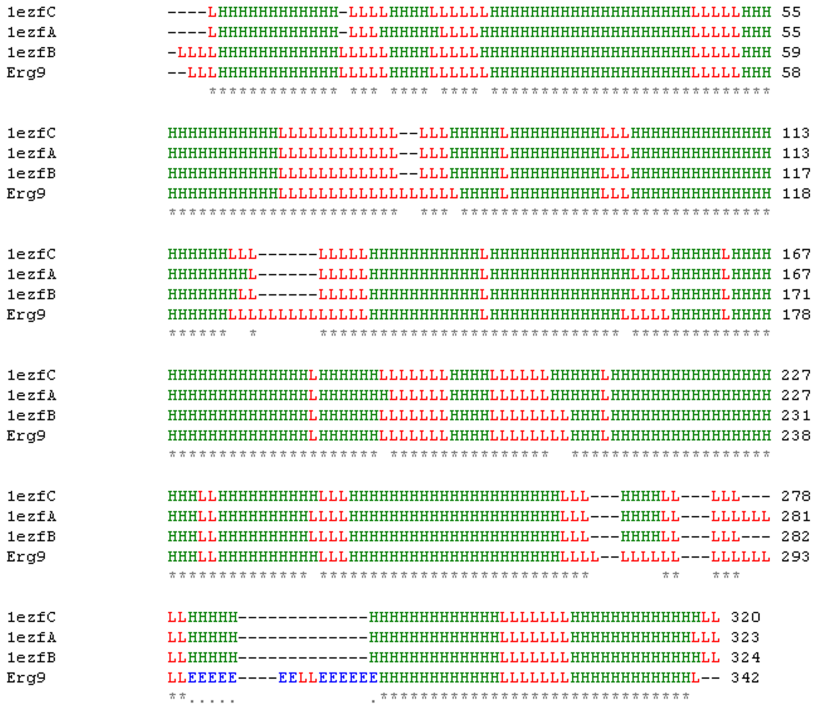


**Figure 3(a):** Secondary structure alignment of *Erg9* protein product with 1ezf A, B and C chains.

| SeqA | Name | SeqB | Name | Score |
| --- | --- | --- | --- | --- |
| 1 | Erg9 | 2 | 1ezfB | 93 |
| 1 | Erg9 | 3 | 1ezfC | 93 |
| 1 | Erg9 | 4 | 1ezfA | 91 |
| 2 | 1ezfB | 3 | 1ezfC | 97 |
| 2 | 1ezfB | 4 | 1ezfA | 95 |
| 3 | 1ezfC | 4 | 1ezfA | 96 |

**Table 4:** Scores generated using ClustalW for secondary structure alignment of *Erg9* protein product against 1ezf A, B and C chains.


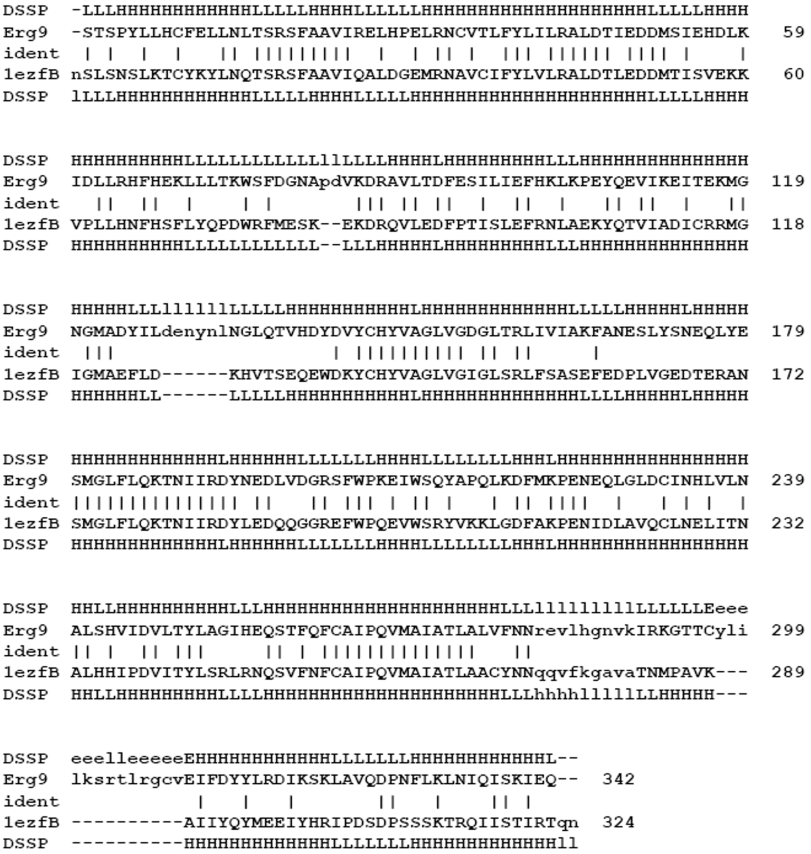


**Figure 3(b):** Pairwise structural alignment of 1ezfB and *Erg9* protein product. Dali server uses DSSP program (Carter *et al*., 2003) for secondary structure assignments from three-dimensional co-ordinates of the given protein structure. Secondary structure Notation: three-state secondary structure definitions by DSSP (H=helix, E=sheet, L=coil) are shown above the amino acid sequence. Structurally equivalent residues are in uppercase, structurally non-equivalent residues (e.g. in loops) are in lowercase. Amino acid identities are marked by vertical bars.

**(a)** **(b)**


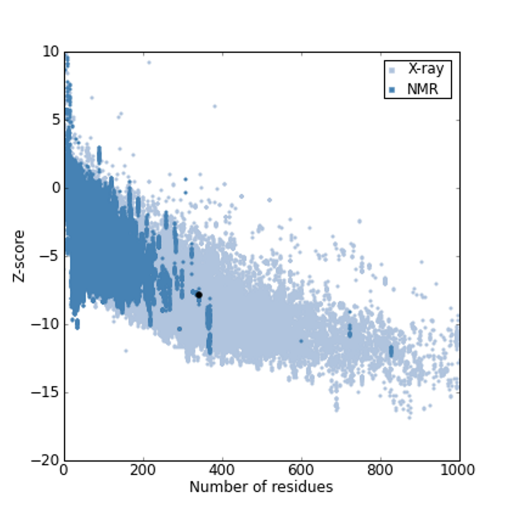

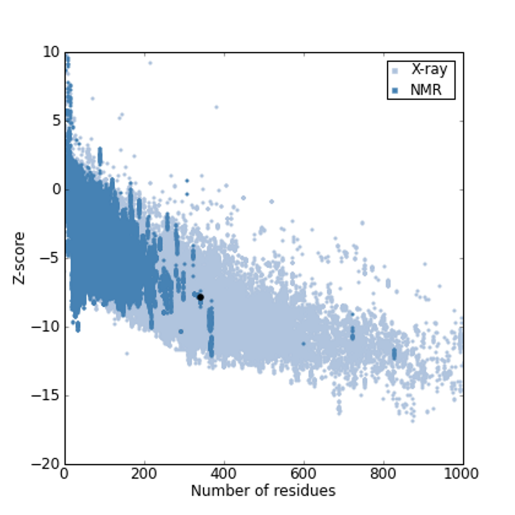


**Figure 4:** Quality assessment of homology models by ProSA-web. (**a)** *Erg9* protein product from S288C. *Z*-Score: -7.88

**(b)** *Erg9* protein product from CEN.PK113-7D. *Z*-Score: -7.85


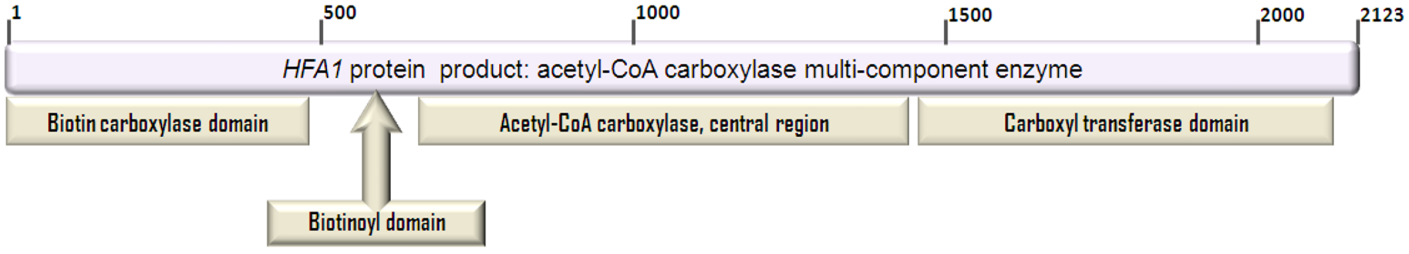


**Figure 5:** Multi domain component system of *HFA1* protein product according to NCBI Conserved Domain Database (Marchler-Bauer *et al*., 2009). Biotin carboxylase is a component of the acetyl-CoA carboxylase multi-component enzyme which catalyses the first committed step in fatty acid synthesis. The biotinoyl domain or biotin carboxyl carrier protein (BCCP) domain is present in all biotin-dependent enzymes and functions in transferring CO2 from one subsite to another, allowing carboxylation, decarboxylation, or transcarboxylation. Acetyl-CoA carboxylase central region featured in this family is found in various eukaryotic acetyl-CoA carboxylases, N-terminal to the catalytic domain. The carboxyl transferase domain carries out the transcarboxylation from biotin to an acceptor molecule.

**Table 5:** Modelled segments of *HFA1* protein product.

| Modeled structure | Template used | PDB name | Modelled Segment of *HFA1* protein product |
| --- | --- | --- | --- |
| 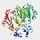 | 1w96A | crystal structure of biotin carboxylase domain of acetyl-coenzyme a carboxylase from *saccharomyces cerevisiae* in complex with soraphen A | 1 - 484 |
| 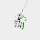 | 2dn8A | solution structure of rsgi ruh-053, an apo-biotin carboxy carrier protein from human transcarboxylase | 608 - 697 |
| 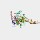 | 1od2A | Crystal structure of the carboxyltransferase domain of acetyl-coenzyme A carboxylase. | 1382 - 2100 |


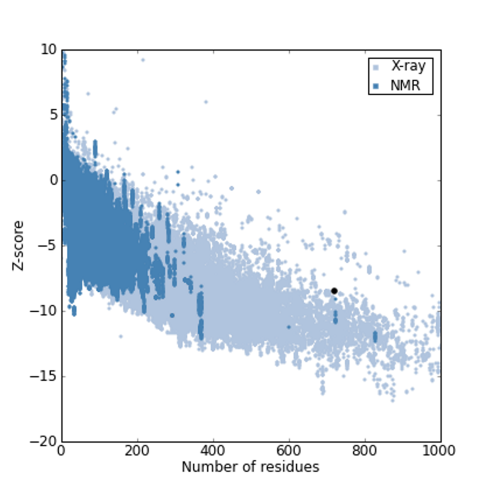

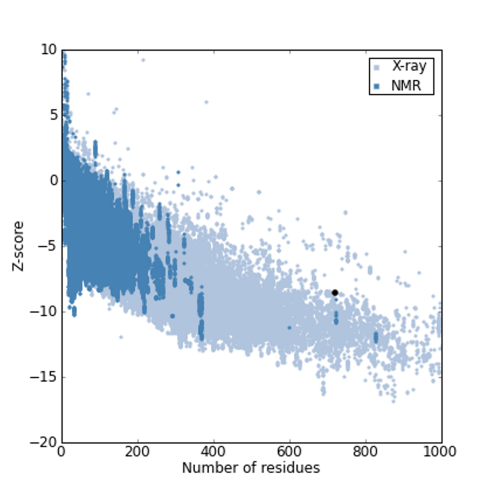


**Figure 6:** Quality assessment of homology models by ProSA-web. (**a)** carboxyl tranferse domain of *HFA1* protein product from S88C. *Z*-Score: -8.51

**(b)** carboxyl tranferse domain of *HFA1* protein product from CEN.PK113-7D. *Z*-Score: -8.53
