# Supplementary material for: Linking Genotype and Phenotype of Saccharomyces cerevisiae Strains Reveals Metabolic Engineering Targets and Leads to Triterpene Hyper-Producers
Source: PLoS One. 2011 Mar 18;6(3):e14763. doi: 10.1371/journal.pone.0014763 (PMC3060802; doi:10.1371/journal.pone.0014763)
Supplement: Supporting Information S2 — Protein stability calculations for Erg8, Erg9 and HFA1 protein products of both S.cerevisiae S288C and S.cerevisiae CEN.PK113-7D strains. Accessible Surface Area (ASA) calculations for Erg8, Erg9 and HFA1 protein products of both S.cerevisiae S288C and S.cerevisiae CEN.PK113-7D RMSD differences between Erg8, Erg9 and HFA1 protein product variants of S.cerevisiae S288C and S.cerevisiae CEN.PK113-7D strains Graph theoretic measures of the structural effects in proteins caused by individual nsSNPs (DOCX) [file pone.0014763.s002.docx]

**Table 1:** Protein stability calculations for Erg8, Erg9 and HFA1 protein products of both *S.cerevisiae* S288C and *S.cerevisiae* CEN.PK113-7D strains.

|  |  |  | ΔΔG (kcal/mol) |
| --- | --- | --- | --- |
|  |  |  |  |
| \| *Erg8*:S288C \| \| --- \| \| | G49E ; S75T ; A192S ; D247N | *Erg8*:CEN.PK113-7D | 9.36 |
|  |  |  |  |
|  |  |  |  |
| \| *Erg8*:CEN.PK113-7D \| \| --- \| \| | E49G ; T75S ; S192A ; N247D | *Erg8*:S288C | -6.32 |
|  |  |  |  |
|  |  |  |  |
|  |  |  |  |
| \| *Erg9*:S288C \| \| --- \| \| | G286S | *Erg9*:CEN.PK113-7D | -37.06 |
|  |  |  |  |
|  |  |  |  |
| \| *Erg9*:CEN.PK113-7D \| \| --- \| \| | S286G | *Erg9*:S288C | >10 |
|  |  |  |  |
|  |  |  |  |
|  |  |  |  |
| \| *HFA1* (CT) :S288C \| \| --- \| \| | I1798T | *HFA1* (CT): CEN.PK113-7D | -1.59 |
|  |  |  |  |
|  |  |  |  |
| \| *HFA1* (CT): CEN.PK113-7D \| \| --- \| \| | T1798I | *HFA1*:S288C | -1.35 |
|  |  |  |  |
|  |  |  |  |

ΔΔG < 0: stabilizing mutations; ΔΔG > 0: destabilizing mutations

*HFA1* (CT) = Carboxyl transferase domain of *HFA1* protein product

**Table 2:** Accessible Surface Area (ASA) calculations for Erg8, Erg9 and HFA1 protein products of both *S.cerevisiae* S288C and *S.cerevisiae* CEN.PK113-7D strains.

|  | POLAR area/energy | APOLAR area/energy | Total area/energy | Number of surface atoms | Number of buried atoms |
| --- | --- | --- | --- | --- | --- |
| *Erg8*:S288C | 7410.72 | 13249.91 | 20660.64 | 1945 | 1231 |
| *Erg8*:CEN.PK113-7D | 7442.29 | 13240.64 | 20682.92 | 1947 | 1236 |
| *Erg9*:S288C | 5873.76 | 9216.47 | 15090.23 | 1427 | 1281 |
| *Erg9*:CEN.PK113-7D | 6408.65 | 9306.87 | 15715.52 | 1417 | 1293 |
| *HFA1*(CT):S288C | 13526.81 | 22554.95 | 36081.77 | 3146 | 2676 |
| *HFA1*(CT):CEN.PK113-7D | 13209.95 | 22928.31 | 36138.26 | 3179 | 2642 |

*HFA1* (CT) = Carboxyl transferase domain of *HFA1* protein product

**Table 3:** RMSD differences between Erg8, *Erg9* and *HFA1* protein product variants *of S.cerevisiae* S288C and *S.cerevisiae* CEN.PK113-7D strains.

|  |  | Alpha Carbons | Back Bone | Heavy | All |
| --- | --- | --- | --- | --- | --- |
| Erg8:S288C & Erg8:CEN.PK113-7D | RMSD | 1.86 | 1.82 | 2.02 | 2.02 |
| Erg9:S288C & Erg8:CEN.PK113-7D | RMSD | 0.01 | 0 | 0 | 0 |
| HFA1(CT):S288C & HFA1(CT):CEN.PK113-7D | RMSD | 1.16 | 1.16 | 1.63 | 1.63 |

**Figure 1:** Residue-residue interaction graphs generated using *Bongo* server. Each circle in the graph is a vertex which represents a residue; each black line is an edge that connects two vertexes, in which case it represents a backbone that links two residues. Notation of secondary structures: the pink vertexes represent the residues in α-helices; the yellow vertexes represent the residues in β-strands and the white vertexes represent the residues in loops. Notation of secondary structure segments: the grey patches indicate the segment of secondary structures (some patches seems to contain residue numbers that are not consecutive, but they actually contain multiple patches which are too close to each other and cannot be separated in the graph). Notation of residue-residue interactions: the blue lines represent hydrogen bonds; the cyan lines represent pi-pi interactions; the purple lines represent pi-cation interactions; the green lines represent hydrophobic interactions around residues that are considered to be involved in hydrophobic cores. The target nsSNP or single point mutation is shown as a red as it is easier to locate it. (a) *Erg8* protein product from *S.cerevisiae* S288C. (b) G49E mutant of *Erg8* protein product. (c) S75T mutant of *Erg8* protein product. (d) A192S mutant of *Erg8* protein product. (e) D247N mutant of *Erg8* protein product.

**8(a)**

**
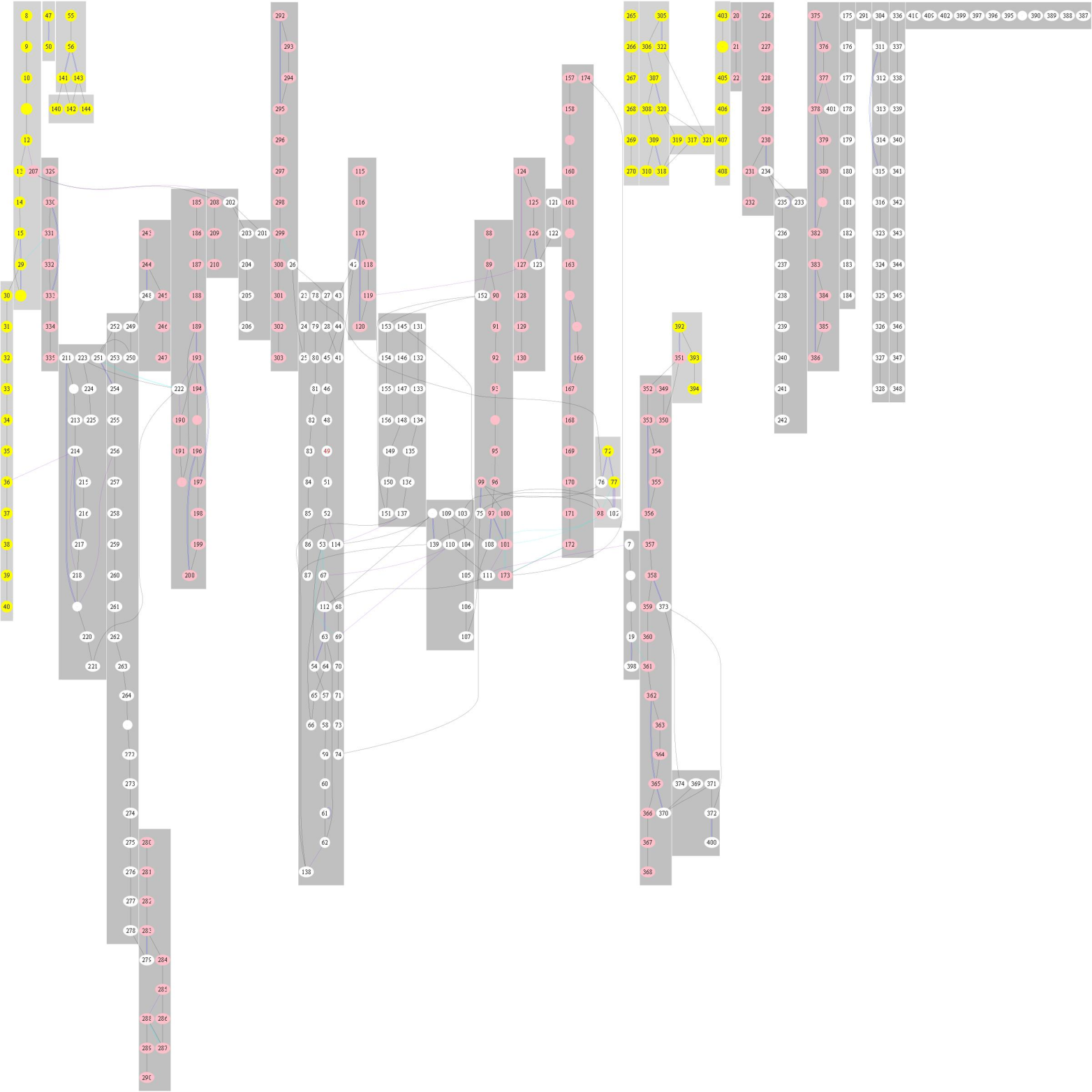
**

**8(b)**

**
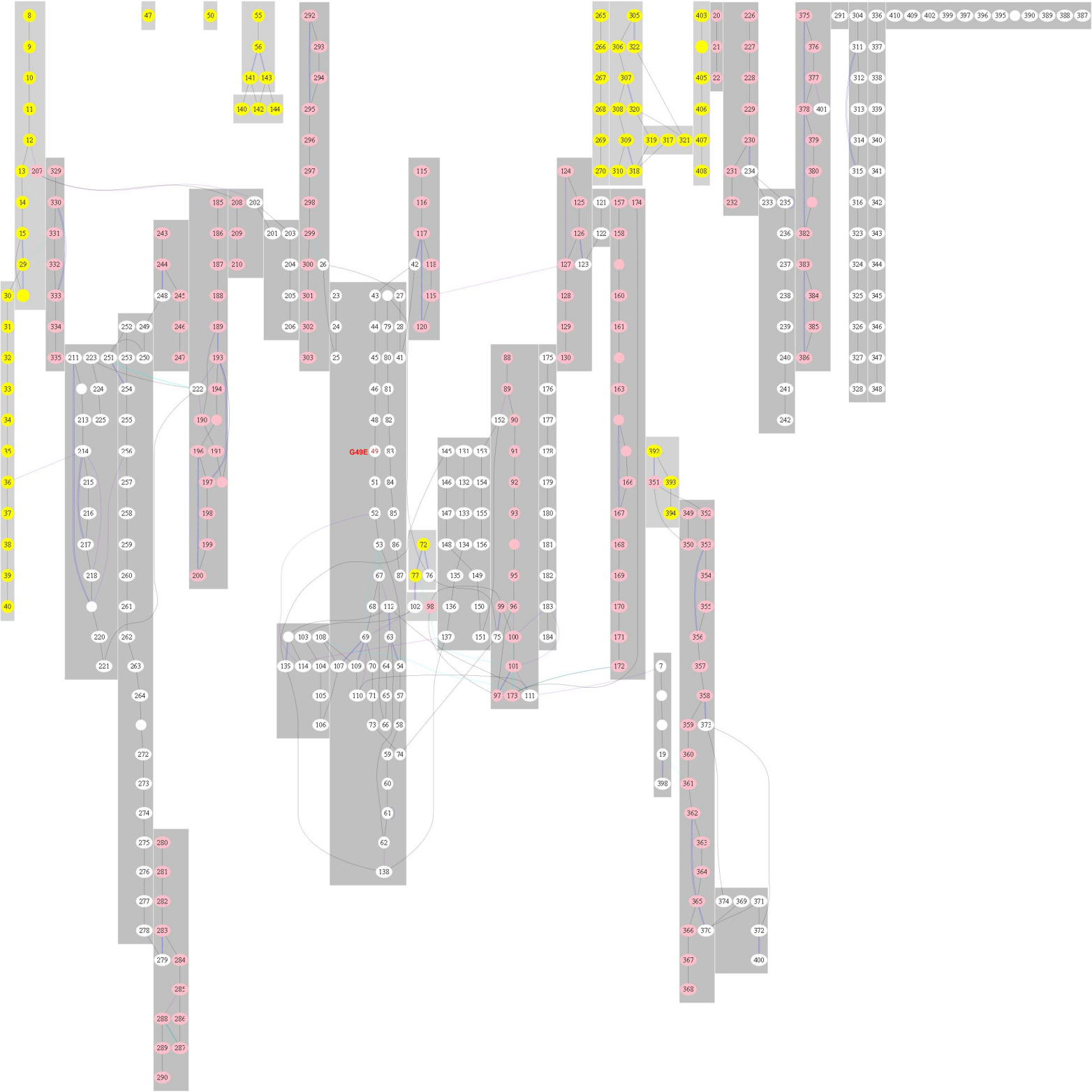
**

**8(c)**

**
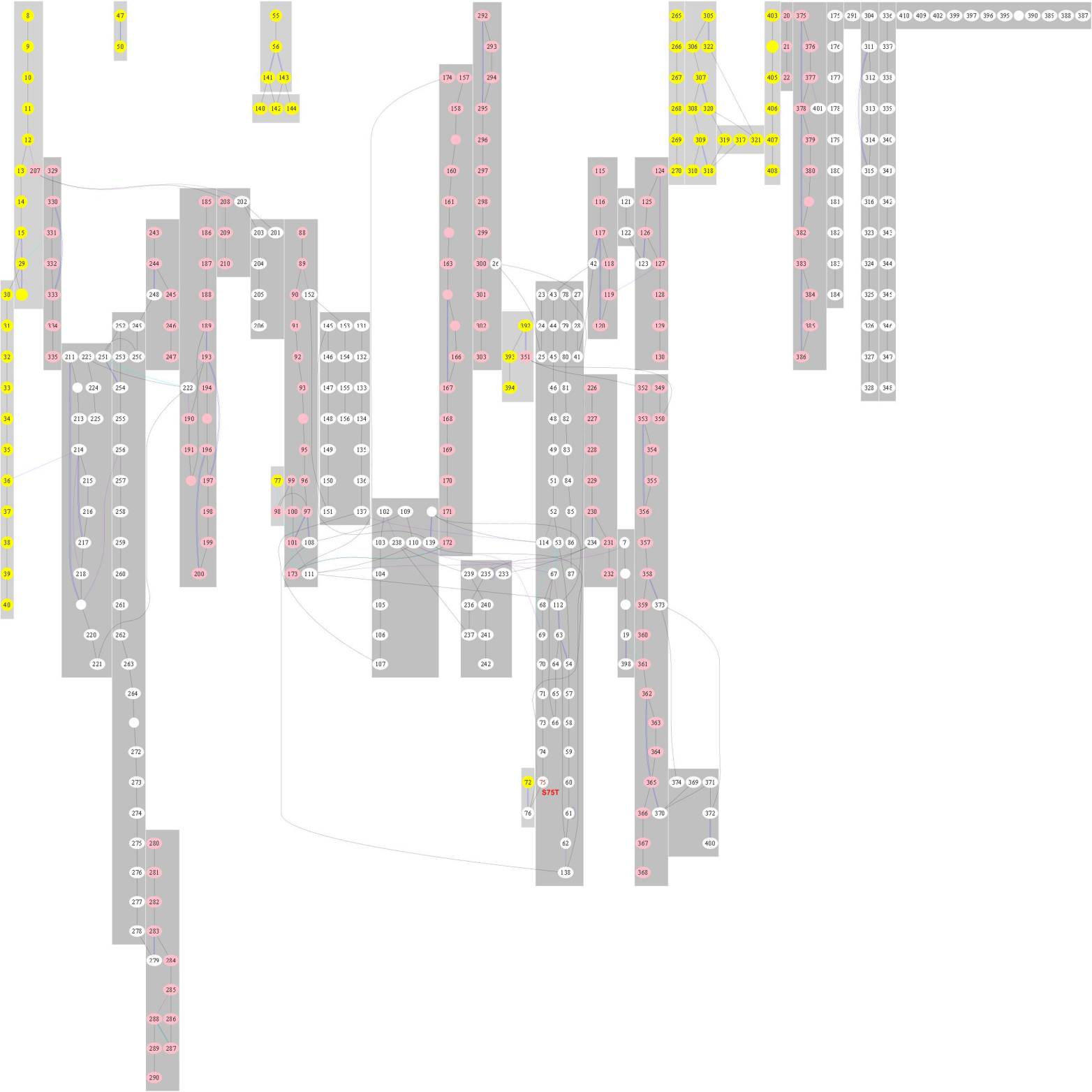
**

**8(d)**

**
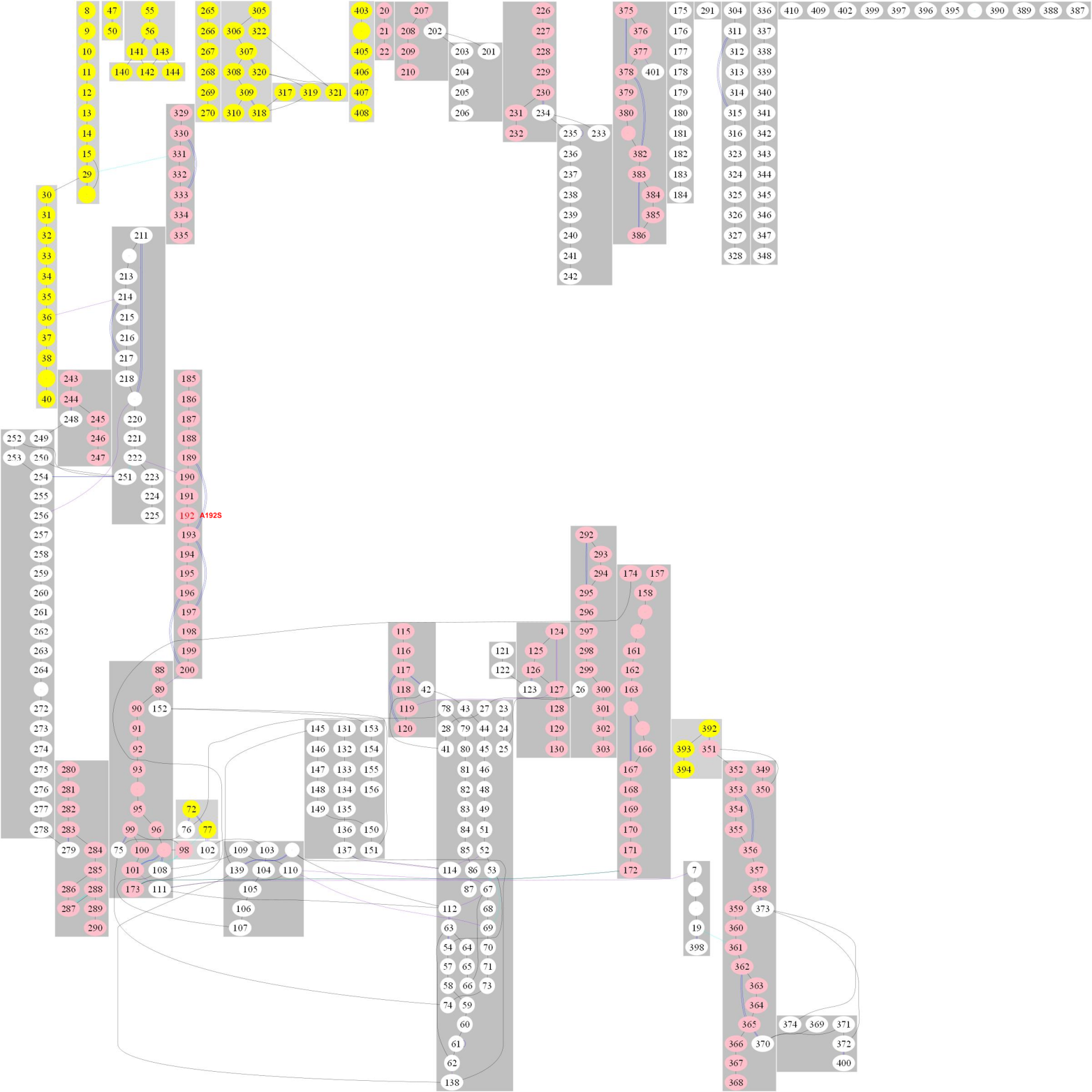
**

**8(e)**

**
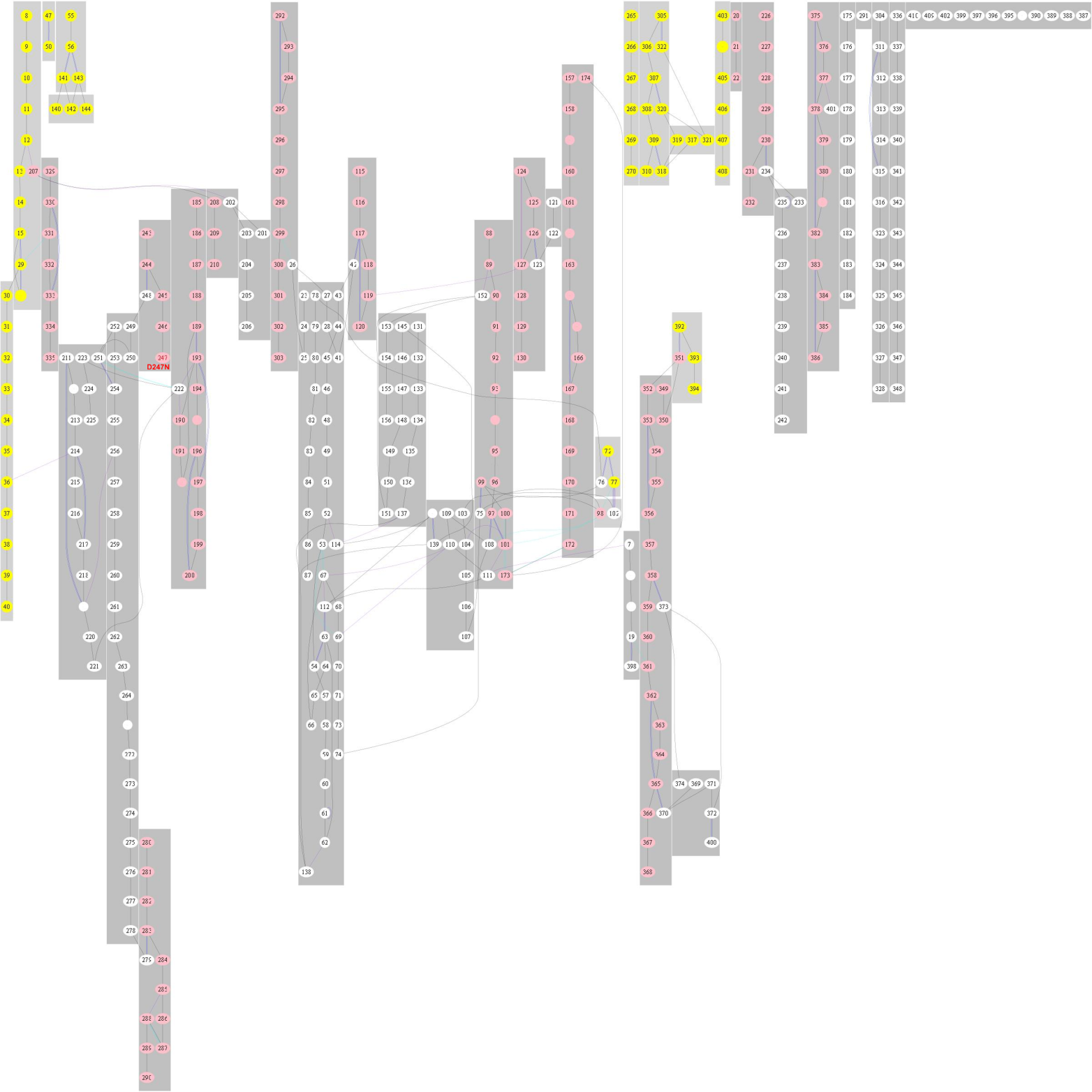
**

**Figure 2:** Residue-residue interaction graphs generated for *Erg9* protein product variants using *Bongo* server. Each circle in the graph is a vertex which represents a residue; each black line is an edge that connects two vertexes, in which case it represents a backbone that links two residues. Notation of secondary structures: the pink vertexes represent the residues in α-helices; the yellow vertexes represent the residues in β-strands and the white vertexes represent the residues in loops. Notation of secondary structure segments: the grey patches indicate the segment of secondary structures (some patches seems to contain residue numbers that are not consecutive, but they actually contain multiple patches which are too close to each other and cannot be separated in the graph). Notation of residue-residue interactions: the blue lines represent hydrogen bonds; the cyan lines represent pi-pi interactions; the purple lines represent pi-cation interactions; the green lines represent hydrophobic interactions around residues that are considered to be involved in hydrophobic cores. The target nsSNP or single point mutation is shown as a red as it is easier to locate it. (a) *Erg9* protein product from *S.cerevisiae* S288C. (b) G286S mutant of *Erg9* protein product. Interestingly, no change in local or global residue-residue interaction networks was observed for G286S *Erg9* mutant.

**(a)**

**
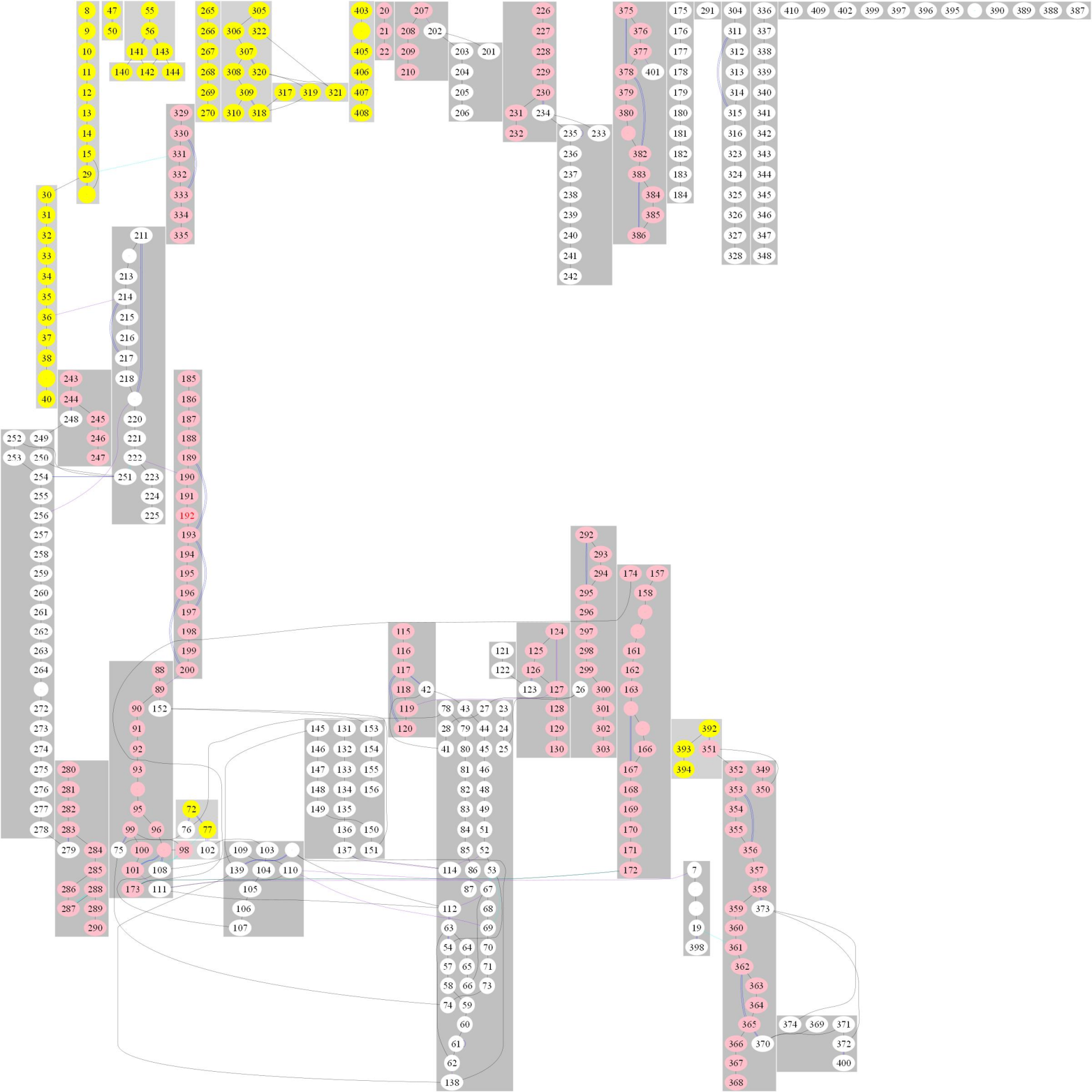
**

**(b)**

**
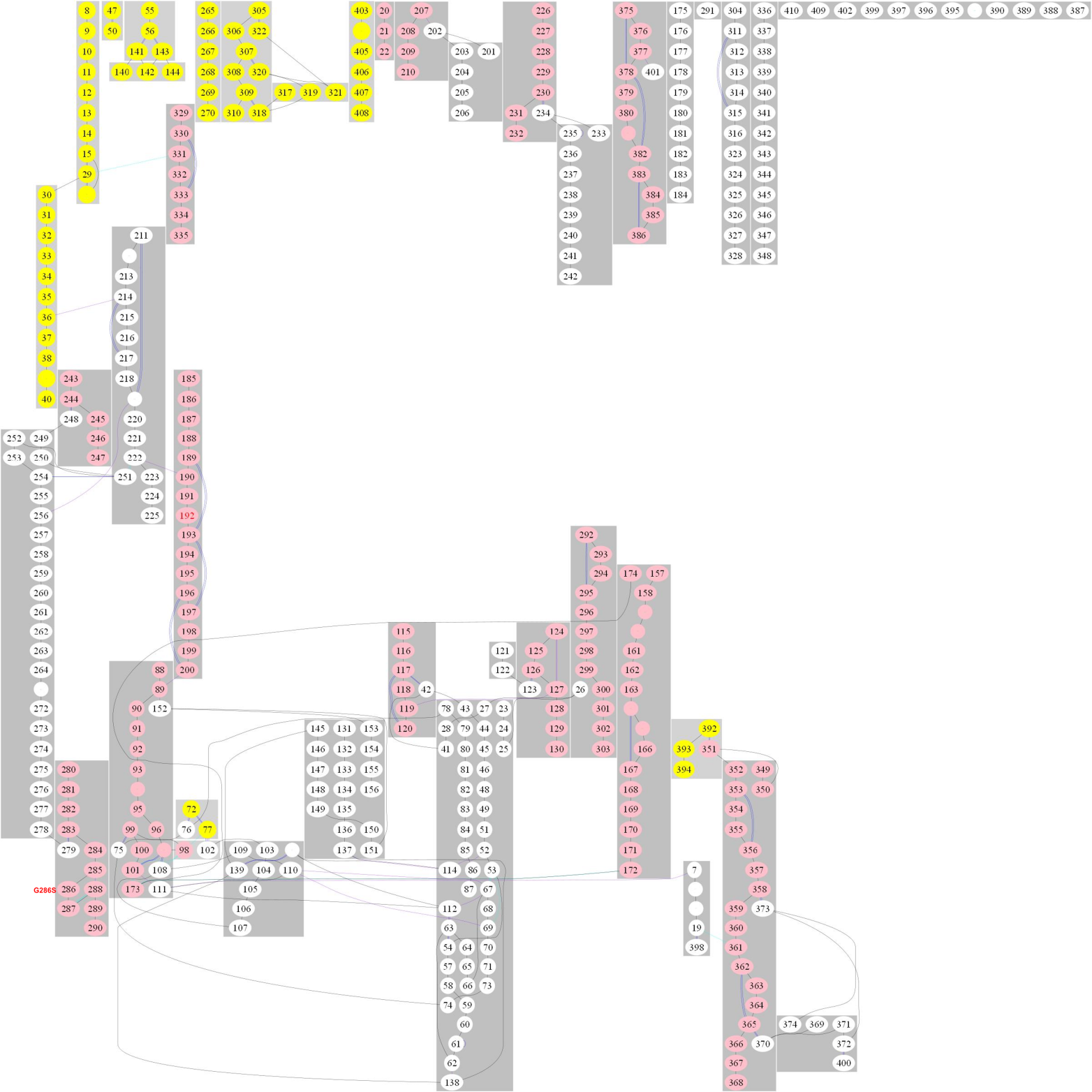
**
