# Supplementary material for: Linking Genotype and Phenotype of Saccharomyces cerevisiae Strains Reveals Metabolic Engineering Targets and Leads to Triterpene Hyper-Producers
Source: PLoS One. 2011 Mar 18;6(3):e14763. doi: 10.1371/journal.pone.0014763 (PMC3060802; doi:10.1371/journal.pone.0014763)
Supplement: Supporting Information S3 — Ligand binding sites of the squalene synthase predicted using the Q-SiteFinder and structural analysis of the carboxyl transferase domain. (PDF) [file pone.0014763.s003.pdf]

Figure 1: Ligand binding sites of squalene synthase predicted using Q-SiteFinder. The two top ranked binding pockets were selected. No difference was observed in the binding pockets of squalene synthase from *S.cerevisiae* S288C and *S.cerevisiae* CEN.PK113-7D strains.

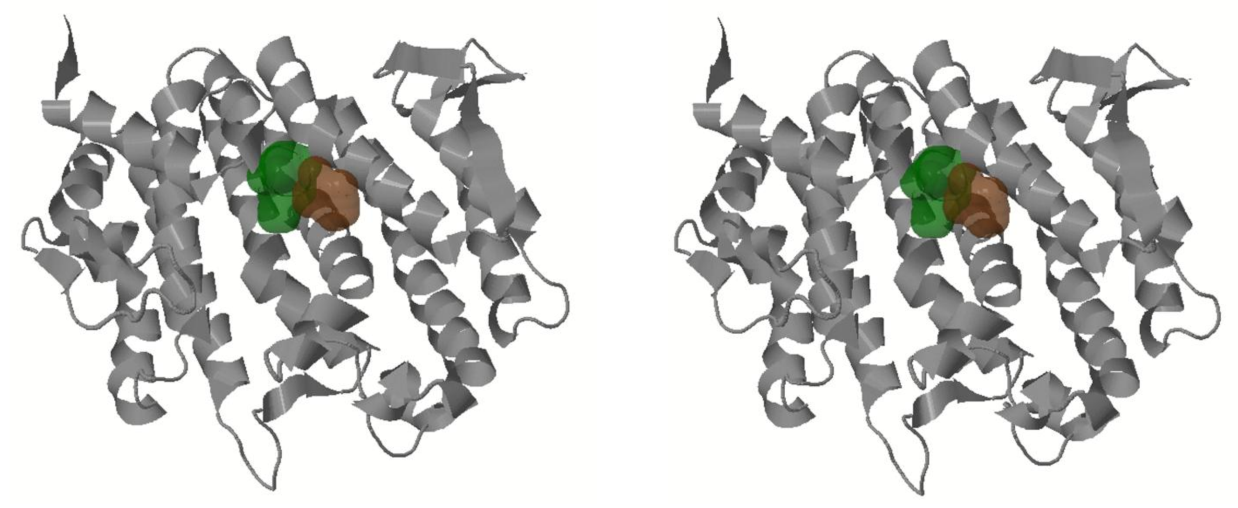

(a) Squalene synthase from S288C strain
Site 1 Volume (cadmium green):
265 Cubic Angstroms
Site 2 Volume (brown):
127 Cubic Angstroms

(b) Squalene synthase from CEN.PK113-7D strain
Site 1 Volume (cadmium green):
265 Cubic Angstroms
Site 2 Volume (brown):
127 Cubic Angstroms

Figure 2: Structural analysis of *HFA1* protein product (Carboxyl transferase domain).

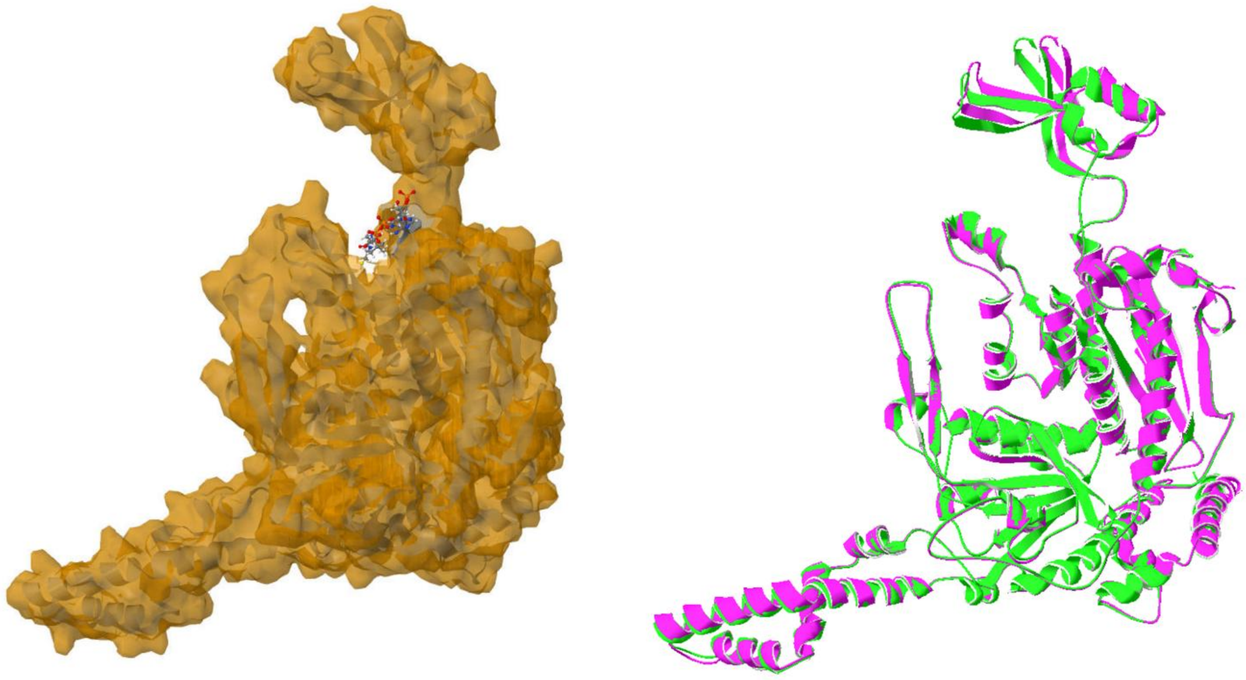

(a) Surface view of crystal structure of yeast carboxyl transferase domain (PDB ID:1OD2) in complex with CoA. The opaque gray ribbon structure is also shown. CoA molecule is represented in ball and stick model. The molecular surface view with opaque ribbon structure was created using Jmol (Herraez, 2006).

(b) Structure superposition of carboxyl transferase domain of HFA1 protein product from *S.cerevisiae* S288C (bright green) and *S.cerevisiae* CEN.PK113-7D (magenta). The changes in the loops and β-sheets at the top of CoA binding pocket can be observed. To locate the CoA binding site see the structure shown in (a).
